# Supplementary material for: Effect of stroke early supported discharge on length of hospital stay: analysis from a national stroke registry
Source: BMJ Open. 2021 Jan 20;11(1):e043480. doi: 10.1136/bmjopen-2020-043480 (PMC7818805; doi:10.1136/bmjopen-2020-043480)
Supplement: Author's manuscript [file bmjopen-2020-043480.draft_revisions.pdf]

BMJ Open is committed to open peer review. As part of this commitment we make the peer review history of every article we publish publicly available.

When an article is published we post the peer reviewers' comments and the authors' responses online. We also post the versions of the paper that were used during peer review. These are the versions that the peer review comments apply to.

The versions of the paper that follow are the versions that were submitted during the peer review process. They are not the versions of record or the final published versions. They should not be cited or distributed as the published version of this manuscript.

BMJ Open is an open access journal and the full, final, typeset and author-corrected version of record of the manuscript is available on our site with no access controls, subscription charges or pay-per-view fees (http://bmjopen.bmj.com).

If you have any questions on BMJ Open's open peer review process please email info.bmjopen@bmj.com

Effect of Stroke Early Supported Discharge on length of hospital stay: Analysis from a National Stroke Registry

Journal:	*BMJ Open*
Manuscript ID	bmjopen-2020-043480
Article Type:	Original research
Date Submitted by the Author:	04-Aug-2020

Complete List of Authors:	Fisher, Rebecca; University of Nottingham<br>Byrne, Adrian; University of Nottingham, Division of Rehabilitation, Ageing and Wellbeing<br>Chouliara, Niki; University of Nottingham<br>Lewis, Sarah; University of Nottingham<br>Paley, Lizz; King's College London<br>Hoffman, Alex; King's College London<br>Rudd, Anthony; King's College London<br>Robinson, Thompson; University of Leicester<br>Langhorne, Peter; University of Glasgow<br>Walker, Marion; University of Nottingham

Keywords:	STROKE MEDICINE, REHABILITATION MEDICINE, Health policy < HEALTH SERVICES ADMINISTRATION & MANAGEMENT, Stroke < NEUROLOGY

SCHOLARONE™ Manuscripts

I, the Submitting Author has the right to grant and does grant on behalf of all authors of the Work (as defined in the below author licence), an exclusive licence and/or a non-exclusive licence for contributions from authors who are: i) UK Crown employees; ii) where BMJ has agreed a CC-BY licence shall apply, and/or iii) in accordance with the terms applicable for US Federal Government officers or employees acting as part of their official duties; on a worldwide, perpetual, irrevocable, royalty-free basis to BMJ Publishing Group Ltd ("BMJ") its licensees and where the relevant Journal is co-owned by BMJ to the co-owners of the Journal, to publish the Work in this journal and any other BMJ products and to exploit all rights, as set out in our licence.

The Submitting Author accepts and understands that any supply made under these terms is made by BMJ to the Submitting Author unless you are acting as an employee on behalf of your employer or a postgraduate student of an affiliated institution which is paying any applicable article publishing charge ("APC") for Open Access articles. Where the Submitting Author wishes to make the Work available on an Open Access basis (and intends to pay the relevant APC), the terms of reuse of such Open Access shall be governed by a Creative Commons licence – details of these licences and which Creative Commons licence will apply to this Work are set out in our licence referred to above.

Other than as permitted in any relevant BMJ Author's Self Archiving Policies, I confirm this Work has not been accepted for publication elsewhere, is not being considered for publication elsewhere and does not duplicate material already published. I confirm all authors consent to publication of this Work and authorise the granting of this licence.

For peer review only

Effect of Stroke Early Supported Discharge on length of hospital stay: Analysis from a National Stroke Registry

First author surname: Fisher

Short title: **Effect of Stroke Early Supported Discharge on length of hospital stay**

Rebecca J Fisher, PhD; Adrian Byrne, PhD; Niki Chouliara, PhD; Sarah Lewis, PhD; Lizz Paley, MSc; Alex Hoffman, MSc; Anthony Rudd, MD; Thompson Robinson, MD; Peter Langhorne, PhD; Marion F Walker, PhD.

University of Nottingham, Nottingham, United Kingdom (RF,AB,NC,SL,MW)
King's College London, London, United Kingdom (LP,AH,AR)
University of Leicester, Leicester, United Kingdom (TR)
University of Glasgow, Glasgow, United Kingdom (PL)

Corresponding author

Dr Rebecca Fisher
Division of Rehabilitation, Ageing & Wellbeing
School of Medicine
University of Nottingham
NG7 2UH

+44 (0) 115 82 30253
rebecca.fisher@nottingham.ac.uk

I, the Submitting Author has the right to grant and does grant on behalf of all authors of the Work (as defined in the below author licence), an exclusive licence and/or a non-exclusive licence for contributions from authors who are: i) UK Crown employees; ii) where BMJ has agreed a CC-BY licence shall apply, and/or iii) in accordance with the terms applicable for US Federal Government officers or employees acting as part of their official duties; on a worldwide, perpetual, irrevocable, royalty-free basis to BMJ Publishing Group Ltd ("BMJ") its licensees and where the relevant Journal is co-owned by BMJ to the co-owners of the Journal, to publish the Work in BMJ Open and any other BMJ products and to exploit all rights, as set out in our licence.

The Submitting Author accepts and understands that any supply made under these terms is made by BMJ to the Submitting Author unless you are acting as an employee on behalf of your employer or a postgraduate student of an affiliated institution which is paying any applicable article publishing charge ("APC") for Open Access articles. Where the Submitting Author wishes to make the Work available on an Open Access basis (and intends to pay the relevant APC), the terms of reuse of such Open Access shall be governed by a Creative Commons licence – details of these licences and which Creative Commons licence will apply to this Work are set out in our licence referred to above.

Key words: early supported discharge, stroke delivery, health services research, patient discharge, rehabilitation, evaluation, health policy

Word count: 3,462

Total number of tables and figures: Tables 5; Figures 1.

For peer review only - http://bmjopen.bmj.com/site/about/guidelines.xhtml

For peer review only

Abstract

Objective − The first observational study to investigate the impact of Early Supported Discharge (ESD) on length of hospital stay in real world conditions.

Design − Using historical prospective Sentinel Stroke National Audit Programme (SSNAP) data (1 Jan 2013 – 31 Dec 2016) and multilevel modelling, cross-sectional (2015-2016; 30,791 patients nested within 55 hospitals) and repeated cross-sectional (2013-2014 vs 2015-2016; 49,266 patients nested within 41 hospitals) were undertaken.

Setting − Hospitals were sampled across a large geographical area of England covering the West and East Midlands, the East of England and the North of England.

Participants – Stroke patients whose data were entered into the SSNAP database by hospital teams.

Interventions − Receiving ESD along the patient care pathway

Primary and secondary outcome measures – Length of hospital stay

Results − When adjusted for important case-mix variables, patients who received ESD on their stroke care pathway spent longer in hospital, compared to those who did not receive ESD. The percentage increase was 15.8% (95% CI 12.3% - 19.4%) for the 2015-2016 cross-sectional analysis and 18.8% (95% CI 13.9% - 24.0%) for the 2013-2014 versus 2015-2016 repeated cross-sectional analysis. On average, the increased length of hospital stay was approximately 1 day.

Conclusions − This study has shown that by comparing ESD and non-ESD patient groups matched for important patient characteristics, receiving ESD resulted in a 1 day increase in length of hospital stay. The large reduction in length of hospital stay overall, since original trials were conducted, may explain why a reduction was not observed. The longer term benefits of accessing ESD need to be investigated further.

Study registration − http://www.isrctn.com/ISRCTN15568163

For peer review only - http://bmjopen.bmj.com/site/about/guidelines.xhtml

Strengths and limitations of this study

- First observational study comparing length of hospital stay in real world settings for patients accessing Early Supported Discharge (ESD) compared to those that did not
- Use of datasets from the Sentinel Stroke National Audit Programme (SSNAP), permitting sample sizes of 30,791 and 49,266 patients
- Accounts for the variation that exists between patients clustered within their admitting hospitals by employing a multilevel modelling approach and reinforces group comparisons using a propensity score matching process
- Unable to account for residual confounding
- Did not investigate the longer term benefits of accessing ESD

For peer review only

For peer review only - http://bmjopen.bmj.com/site/about/guidelines.xhtml

Introduction

Stroke is one of the main causes of adult disability and there is strong research evidence that provision of stroke specialist rehabilitation enhances recovery.[1] Stroke Early Supported Discharge (ESD) is a multidisciplinary team intervention aimed at facilitating discharge from hospital and providing stroke specialist rehabilitation at home.[2] Based on cumulative evidence from clinical trials, stroke care guidelines in the UK and worldwide, recommend the provision of ESD as part of an evidence-based stroke care pathway.[3-8]

In many high-income countries, ESD has not been well developed in practice with a lack of large scale implementation.[9] In contrast, in the UK, considerable efforts have been made to implement ESD, although types of service differ across the country, and in some regions, ESD is still not offered at all.[10] Where ESD has been delivered, it is unclear whether benefits of the ESD intervention, as suggested in clinical trials, are achieved in practice.

One of the attractive features of the ESD intervention as reported in clinical trials, has been reduction in length of hospital stay, with the most recent Cochrane systematic review suggesting a reduction of 6 days.[2] This reduction has contributed to the reported cost-effectiveness of combined stroke unit care and ESD.[11] Small scale evaluation of ESD operating in localised areas have suggested smaller, but significant, reductions in length of acute hospital stay, but it remains unknown what impact ESD is having over a larger scale.[12] Average length of hospital stay for all stroke patients discharged alive fell from 40 days in 2001 to 20 days in 2013 and has remained around this level since then.[13] This trend over time has meant more stroke unit beds are available for new patients but it has also increased demand for patient care in the community post hospital discharge.

The Sentinel Stroke National Audit programme (SSNAP) is the national stroke register of England, Wales and Northern Ireland in which all acute admitting hospitals and post-acute

For peer review only - http://bmjopen.bmj.com/site/about/guidelines.xhtml

stroke teams are mandated to participate.[14] SSNAP has played a key role in monitoring performance and improving provision of acute stroke care. Collection of SSNAP data across the stroke care pathway now offers a unique opportunity to investigate the large scale impact of post-acute interventions such as ESD. The study reported here, and in a parallel paper exploring the effectiveness of ESD service models, investigates if trial-based benefits of ESD are realised in practice, with the aim to address inequality and inform evidence based improvements.[15] Here, we focus on the impact of ESD on length of hospital stay.

Methods

Study design

We present results from an observational cohort study (Figure 1), conducted as part of an overall mixed method study.[16] The study protocol was approved by a University of Nottingham Ethics Committee and the UK Healthcare Quality Improvement Partnership Data Access Request Group. Data access requests should be directed to Healthcare Quality Improvement Partnership as the data controller and SSNAP as the data provider. The study protocol including statistical analysis plan is available online.[16] We determined a priori, a sample size of 21,760 patients for a study power of 80% to detect standardised effect sizes of 0.25 for each outcome.

In this paper, we have used two different study designs: (1) a cross-sectional analysis (SSNAP data from 2015-2016 time period) comparing length of hospital stay between patients who did and did not have ESD on their care pathway, with adjustment for hospital and individual confounding factors; (2) repeated cross-sectional analysis using two sets of

For peer review only - http://bmjopen.bmj.com/site/about/guidelines.xhtml

SSNAP data (2013-2014 and 2015-2016) to establish whether length of hospital stay changed over time and whether any change was attributable to ESD.

Setting

Hospitals (and associated ESD services) were sampled across a large geographical area of England. The sampling strategy was devised in accordance with the overall mixed method study design and included all ESD services in specific regions of England.[16] Here we report findings from the quantitative investigation of ESD effect on length of hospital stay across West and East Midlands and East of England, (across which a specific initiative to promote ESD was initiated in 2010) and the North of England, a region with a defined lack of ESD.[10,17] We have included all main referring hospitals within the defined geographical area as well as referring hospitals outside of that catchment area whose patients did and did not go onto receive ESD along their care pathway. Those that did receive ESD did so within the catchment area.

Data sources & participants

Patient-level SSNAP data are entered by clinical teams onto a secure webtool with real time data validations to ensure data quality.[14] Historical prospective clinical (patient-level) SSNAP data were obtained from the SSNAP team with permission from Healthcare Quality Improvement Partnership. The first study design involved 55 admitting hospitals and SSNAP data for all patients admitted during 1 Sep 2015 – 31 Dec 2016 (31,556 stroke patients). The second study design included 41 admitting hospitals and SSNAP data for all patients admitted during two time periods, 1 Sep 2013 – 31 Dec 2014 and 1 Sep 2015 – 31 Dec 2016 (50,793 stroke patients).

For peer review only - http://bmjopen.bmj.com/site/about/guidelines.xhtml

Outcomes

Length of hospital stay was defined as the total length of hospital stay per patient (as an inpatient) from arrival at the admitting hospital (or time of stroke onset, if already an inpatient), to the time of discharge from the last inpatient setting, including time spent at any other inpatient setting. Owing to the positive skewness of the underlying distribution, length of hospital stay was natural log transformed before undertaking the statistical modelling and all effect sizes were exponentiated afterwards to aid interpretation.

Confounding factors

In order to investigate the effect of ESD on length of hospital stay, we identified a need to control for the overall standard of hospital care and also the influence of provision of social care. At the hospital level we included two confounding variables: a hospital SSNAP rating score and a measure of delayed transfers of care from hospital, derived from the Adult Social Care Outcomes Framework (ASCOF).

The hospital rating scores used in this study were an overall quality rating for each hospital obtained from SSNAP (total key indicator score derived across ten domains of stroke care with adjustments made for case ascertainment levels and the quality of data submitted to SSNAP).[18] SSNAP produced performance rating scores for admitting hospitals during the periods of interest and we used the score produced during the latter part of the period of interest for each analysis.[19] This score for each admitting hospital was used as an indication of the overall standard of inpatient care after the onset of stroke. The ASCOF data reports the average daily rate of delayed transfers of care per 100,000 population aged 18 and over at local authority level. Data were aggregated up to National Health Service Trust level, using averages where multiple local authorities were associated with one National Health Service

For peer review only - http://bmjopen.bmj.com/site/about/guidelines.xhtml

Trust. Again we focused on the scores produced during the latter part of the period of interest in line with the SSNAP hospital performance ratings.[20]

In order to account for comparison between different groups of individual patients, we also included variables at the patient level. These were stroke patient characteristics, reflecting validated stroke case-mix models and collected as part of the SSNAP data set. These were age at admission, sex, pre-stroke independence, cardiovascular comorbidities, NIH stroke scale score on admission, type of stroke and modified Rankin score at discharge from hospital.[21,22]

Statistical analyses

We employed a linear multilevel model framework for both study designs whereby patients (level 1) were clustered within admitting hospitals (level 2) in an approach consistent with previous observational studies of this type.[15,21-24] Within our multilevel models, we adjusted for a range of patient and hospital characteristics as covariates. We chose multilevel modelling to evaluate the effect of ESD on length of hospital stay as it could accommodate and appreciate the variation that existed within and between different admitting hospitals. Furthermore, the intraclass correlation coefficient was calculated as a measure of proportion of the total variance in length of hospital stay, which was attributable to variance within admitting hospitals as opposed to between admitting hospitals.

The adequacy of different statistical models was compared using the log-likelihood, Akaike Information Criterion and Bayesian Information Criterion values from single level and multilevel regression models with multilevel preferable on each occasion. Multicollinearity was investigated by examining variance inflation factor scores of all predictor variable sets and was found not to be an issue. Covariate linearity was examined by checking the consistency of a linear trend in relation to each outcome variable. Regarding the impact of

For peer review only - http://bmjopen.bmj.com/site/about/guidelines.xhtml

missing data, we assessed the mean change in the outcome variable between the ESD and non-ESD groups in relation to missing predictor data (which amounted to a loss of 0.6% of the total sample size); no substantial differences were found as we observed a mean difference of less than 0.3%.

Analysis of cross-sectional data from 2015-2016

A quasi-experimental cross-sectional design was used in which patients who received ESD on their care pathway were compared with patients that had not (non-ESD). Hence across 55 admitting hospitals covering the 1 Sep 2015 – 31 Dec 2016 data period, we investigated the effect of ESD being on the care pathway (yes/no) for stroke patients on their total length of hospital stay from admitting hospital until being discharged from the last inpatient setting.

Analysis of repeated cross-sectional data from 2013-2014 and 2015-2016

This analysis was to establish whether length of hospital stay changed over time i.e. between the earlier (1 Sep 2013 – 31 Dec 2014) and later time periods (1 Sep 2015 – 31 Dec 2016), and whether any change was attributable to ESD, as ESD provision was higher in the later time period.[10] Patients formed distinct groups over the two time periods as they were either admitted in the 2013-2014 time period or the 2015-2016 time period with no patient covering both time periods.[25] The same multilevel model approach was used as in the cross-sectional analysis, but also incorporating an effect of time (13/14 or 15/16), and an interaction between time and the effect of ESD which modelled whether the effect of ESD was similar in the two time periods. The main effect of receiving ESD (with adjustment for time) indicated the effect of ESD on length of hospital stay in the first time period. An interaction between time and ESD would indicate whether there was any difference in the effect of ESD on length of hospital stay between the first and second time periods.

For peer review only - http://bmjopen.bmj.com/site/about/guidelines.xhtml

Sensitivity analysis using propensity score matching

Since it was important to ensure that the effects of ESD were not the result of potential confounding factors, we also used an alternative method of controlling for confounders, i.e. propensity score matching, as a sensitivity analysis. Propensity score matching involved matching patients with ESD on their pathway with similar patients who did not, based on their patient characteristics. In this case, we explored two alternative approaches to propensity score matching, firstly matching patients with ESD to patients without ESD in the same hospital, i.e. within-admitting hospital matching.[26] This resulted in a truncated sample size as only hospitals with both ESD and non-ESD patients could contribute to this first matching analysis.

Secondly, we then allowed the matching to occur with similar patients without ESD in different hospitals, i.e. between-admitting hospital matching.[27] This second approach enabled us to use a larger sample size as all hospitals could contribute to the probability estimate of whether a patient received ESD or not regardless of what their patients actually received. In order to ensure greater accuracy in the latter analysis, we used a stratified matching process so that patients were only matched to patients in a hospital with a similar proportion of patients having access to ESD.

A two tailed significance level of 0.05 was used in all hypothesis tests. We carried out the majority of analyses using Stata/SE 15.1 (Statacorp) and the R package Matching was used to undertake the between-admitting hospital propensity score matching.

Results

Analysis of cross-sectional data from 2015-2016

For peer review only - http://bmjopen.bmj.com/site/about/guidelines.xhtml

Table 1 presents descriptive statistics of the patient and admitting hospital level variables and the median number of days spent as an inpatient disaggregated by patients who received ESD (ESD; n=8648) or not (non-ESD; n=22908) on the care pathway. Compared to patients who did not receive ESD, those who did were younger and more likely to be independent (lower pre-morbid modified Rankin Scale score) before stroke as well as more independent at inpatient discharge (lower post-morbid modified Rankin Scale score). ESD patients were also more likely to have been admitted to a hospital with a higher SSNAP score rating and higher rate of delayed transfers.

Before adjustment, ESD patients had a longer length of hospital stay than non-ESD patients; median 6.9 (interquartile range: 2.9-18.8) compared to 6.0 days (interquartile range: 2.2-24.8). Following multilevel model analysis, after controlling for patient and hospital level characteristics, the mean length of stay for patients who received ESD along their care pathway compared to those who did not remained significantly increased by 15.8% (95% CI 12.3% - 19.4%) (Table 2). Similarly, from the sensitivity analysis using propensity score matching, patients who received ESD on their care pathway, on average, stayed in hospital longer (when controlling for all other patient characteristics, and hospital SSNAP and ASCOF scores) (Table 3). The within-admitting hospital propensity score matching process (n=10449) suggested ESD patients stayed in hospital longer by an average of 8.7% (95% CI 4.8% – 12.6%). The between-admitting hospital propensity score matching process (n=30791) found that ESD patients stayed in hospital longer by an average of 7.3% (95% CI 3.0% – 11.8%).

Analysis of repeated cross-sectional data from 2013-2014 and 2015-2016

Table 4 presents descriptive statistics of the patient and admitting hospital level variables used in the repeated cross-sectional analysis, as well as the median number of days spent as

For peer review only - http://bmjopen.bmj.com/site/about/guidelines.xhtml

an inpatient disaggregated by patients who received ESD (n=11376) or not (non-ESD; n=39417) on the care pathway across the two time periods. This descriptive repeated cross-sectional analysis showed that the numbers of patients who received ESD significantly increased over the two time periods. Moreover, across the two time periods, ESD patients were younger, more likely to be male and more likely to have pre-morbid independence, as evidenced by a lower modified Rankin Scale score. Before adjustment, ESD patients were recorded as having a longer length of stay in hospital than non-ESD patients; median 7.7 (interquartile range: 3.2-23.8) compared to 6.3 days (interquartile range: 2.5-22.1) in 2013-2014, and 6.8 (interquartile range: 2.9-18.5) compared to 5.7 days (interquartile range: 2.1-24.6) in 2015-2016.

Table 5 presents the results from the linear multilevel model. Again, we controlled for patient characteristics, SSNAP admitting hospital score and average ASCOF rates for each admitting hospital. There was no significant interaction between receiving ESD and time period (p = 0.79), so that the effect of ESD was similar in both time periods, despite more patients accessing ESD over time. Therefore, the main effect of ESD across both time periods was estimated to increase length of hospital stay by an average of 18.8% (95% CI 13.9% - 24.0%) compared to patients who did not receive ESD on their care pathway. Furthermore, the main effect of the 2015-2016 time period compared to the 2013-2014 time period was found to significantly reduce the length of hospital stay by an average of 14.0% (95% CI 12.2% - 15.9%).

The degree of clustering was low in both adjusted multilevel models (adjusted intraclass correlation coefficients respectively, 0.08, 0.08) implying that the total variance we observed in the length of hospital stay data was more greatly accounted for by the patients than by the admitting hospitals.

For peer review only - http://bmjopen.bmj.com/site/about/guidelines.xhtml

Discussion

This study has investigated the effect of ESD on length of hospital stay in real world settings, with the aim to address inequality and inform evidence based improvements in stroke care. Unadjusted findings indicated that stroke patients who received ESD spent approximately one day longer in hospital than all other patients, however, those who received ESD differed in characteristics from those who did not. With adjustment using two different designs and alternative analyses for the differing case-mix, length of hospital stay remained longer for those who received ESD on their care pathway. This contrasts with findings from original ESD randomised controlled trials, in which a reduction in length of hospital stay was reported.[2]

This is an important finding, particularly when considering implementation of ESD in practice. The promise of reducing length of hospital stay made ESD attractive with regard to cost savings and is likely to have contributed to widespread implementation in England.[10,11] The economic implications of ESD in the real world have previously been investigated through simulation modelling (informed by SSNAP data similar to data used here).[28] The authors reported an estimated mean length of hospital stay for ESD patients that was 8 days less than non-ESD patients and thereby derived cost savings associated with modelling increased use of ESD.

However, an important difference with that economic analysis and the study reported here relates to the fact that we used actual and total inpatient length of stay data combining different inpatient settings where applicable on a per patient basis. Analysis showed that ESD patients were younger and more likely to have a lower modified Rankin score at inpatient discharge. The simulation modelling findings drew upon sample statistics derived from

For peer review only - http://bmjopen.bmj.com/site/about/guidelines.xhtml

patient distributions for each separate inpatient setting stratified by age category and modified Rankin Scale score and their results were driven by assuming more severe stroke survivors received ESD. What our study adds is that when comparing ESD and non-ESD patient groups matched for important patient characteristics, receiving ESD does not relate to a reduction in length of hospital stay.

Another important consideration is that average length of hospital stay for stroke survivors overall has reduced dramatically over the last twenty years, the latter decade of which our study captures between the two cohorts in this paper (i.e. 2013-2014 and 2015-2016).[13] This makes it less likely for an intervention like ESD to have an effect (in comparison to the years 1997-2004 when the original UK clinical trials were conducted).[2] That ESD in this study was associated with an average increase in length of stay of 1 day, could be interpreted as the need for additional time to facilitate the transfer of care to the ESD team, rather than stroke survivors simply leaving hospital without support (if ESD was not available). Previous studies have also highlighted transfer problems relating to lack of joint working between health and social care.[29-31] Our parallel paper added to this debate by highlighting the importance of access to a social worker as part of the ESD team.[15]

What was not possible to investigate in this study was whether access to ESD (despite an additional day in hospital) was associated with improved patient outcomes over the longer term. Recent observational studies in Sweden suggest patient and care-giver benefits related to provision of ESD in regular clinical practice, in line with our previous study findings from England.[32,33] We suggest routine collection of additional validated patient outcome measures (e.g. measuring activities of daily living, general health/mood and quality of life) at longer follow-up periods in national stroke audits or registries is required.[2,34,35]

For peer review only - http://bmjopen.bmj.com/site/about/guidelines.xhtml

Like other observational studies, this study had its limitations. Although numbers of patients were sufficient for the analysis undertaken, by focusing on specific regions of England, transferability of findings could be questioned, particularly outside England. Other limitations include potential sources of residual confounding and also reliance on SSNAP data being accurately reported by hospital teams. Length of hospital stay data is inherently variable and whilst we tried to include a proxy measure for other sources of delays in discharge, analysis would have benefitted from social care related patient level variables.[28,32] Patients also experienced a series of hospital stays (e.g. transfer between acute and rehabilitation wards), relying on accurate transfer of SSNAP records between hospitals and resulting in us focusing on total length of hospital stay and ESD provision on a care pathway.[36] What this means is that the impact of ESD on a particular hospital may not be apparent, which may be of more interest to providers of hospital services.

Conclusion

Original clinical trials of ESD were conducted across the world and implementation of ESD is recommended in many countries' stroke guidelines.[4-9] This study investigated the impact of ESD in real world settings and focused on length of hospital stay. Whilst ESD was not associated with a reduction in length of hospital stay (as previously reported in clinical trials), the increased average length of stay was just 1 day. What remains to be investigated and reported are the benefits of accessing ESD over the longer term. Also by focusing analysis on patients who would have been eligible, but did not receive ESD, we have highlighted an important gap in service provision.

For peer review only - http://bmjopen.bmj.com/site/about/guidelines.xhtml

Acknowledgments

Fisher (Principal Investigator), Byrne, Lewis contributed to study design, data analysis and interpretation, and article writing; Chouliara, Walker, Robinson, Langhorne contributed to study design and article writing; Paley, Hoffman, Rudd are Sentinel Stroke National Audit Programme (SSNAP) collaborators. We thank the many people and organizations participating in SSNAP and members of the SSNAP collaboration.

Sources of Funding

This research was funded by the National Institute for Health Research (NIHR), Health Services and Delivery Research (HS&DR) Programme (16/01/17). Rebecca Fisher is funded by the Stroke Association (TSA LECT 2016/01 Stroke Association HRH the Princess Margaret Senior Lecturer Award). Thompson Robinson is an NIHR Senior Investigator. The Sentinel Stroke National Audit Programme is commissioned by the Healthcare Quality Improvement Partnership and funded by National Health Service (NHS) England and the Welsh Government.

For peer review only

Disclosures

The views expressed are those of the authors and not necessarily those of the NHS, the NIHR or the Department of Health and Social Care.

Competing interests

All authors declare they have no competing interests.

Patient consent

Not required.

Data sharing agreement

For peer review only - http://bmjopen.bmj.com/site/about/guidelines.xhtml

Data access requests should be directed to Healthcare Quality Improvement Partnership as the data controller and SSNAP as the data provider.

References

1. Langhorne P, Bernhardt J, Kwakkel G. Stroke rehabilitation. The Lancet. 2011; 377:1693–702.

2. Langhorne P, Baylan S. Early Supported Discharge Trialists. Early supported discharge services for people with acute stroke. Cochrane Database Syst Rev. 2017; 7:CD000443.

3. Intercollegiate Stroke Working Party. National clinical guideline for stroke. 5th ed. London: Royal College of Physicians. 2016.

4. The National Institute for Health and Care Excellence. Stroke rehabilitation in adults. NICE guidelines (clinical guideline CG 162). 2013.

5. The European Stroke Organization (ESO) Executive Committee and the ESO Writing Committee. Guidelines for Management of Ischaemic Stroke and Transient Ischaemic Attack 2008. http://www.congrex-switzerland.com/fileadmin/files/2013/eso-stroke/pdf/ESO08_Guidelines_Original_english.pdf. 2008; Accessed 28 Aug 2019.

6. Adeoye O, Nyström KV, Yavagal DR, Luciano J, Nogueira RG, Zorowitz RD, Khalessi AA, Bushnell C, Barsan WG, Panagos P, Alberts MJ, Tiner AC, Schwamm LH, Jauch EC. Recommendations for the Establishment of Stroke Systems of Care: A 2019 Update. A policy statement from the American Stroke Association. Stroke. 2019; 50:e187-e210.

7. Hebert D, Lindsay MP, McIntyre A, Kirton A, Rumney PG, Bagg S, Bayley M, Dowlatshahi D, Dukelow S, Garnhum M, Glasser E, Halabi ML, Kang E, MacKay-Lyons M, Martino R, Rochette A, Rowe S, Salbach N, Semenko B, Stack B, Swinton L, Weber V, Mayer M, Verrilli S, DeVeber G, Andersen J, Barlow K, Cassidy C, Dilenge ME, Fehlings D, Hung R, Iruthayarajah J, Lenz L, Majnemer A, Purtzki J, Rafay M, Sonnenberg LK, Townley A, Janzen S, Foley N, Teasell R. Canadian stroke best practice recommendations: Stroke rehabilitation practice guidelines, update 2015. Int J Stroke. 2016; 11:459-484.

8. Stroke Foundation. Clinical Guidelines for Stroke Management. Melbourne Australia. https://informme.org.au/en/Guidelines/Clinical-Guidelines-for-Stroke-Management. 2019; Accessed 28 Aug 2019.

9. Kings College London for the Stroke Alliance for Europe. The Burden of stroke in Europe report. https://strokeeurope.eu/index/rehabilitation-and-long-term-support/5-4early-supported-discharge/ 2017; Accessed 28 Aug 2019.

10. Sentinel Stroke National Audit Programme (SSNAP). Post-acute organisational audit. Phase 2: Organisational audit of post-acute stroke service providers. https://www.strokeaudit.org/results/Organisational.aspx. 2015; Accessed 15 Mar 2019.

11. Saka O, Serra V, Samyshkin Y, McGuire A, Wolfe C. Cost-Effectiveness of Stroke Unit Care Followed by Early Supported Discharge. Stroke. 2009; 40:24-29.

For peer review only - http://bmjopen.bmj.com/site/about/guidelines.xhtml

12. Neale S, Leach K, Steinfort S, Hitch D. Costs and length of stay associated with early supported discharge for moderate and severe stroke survivors. Journal of Stroke and Cerebrovascular Diseases. 2020; 29:e104996. doi:10.1016/j.jstrokecerebrovasdis.2020.104996

13. Sentinel Stroke National Audit Programme. National Clinical results. https://www.strokeaudit.org/results/Clinical-audit/National-Results.aspx. 2018-2019; Accessed 8 Jun 2020.

14. Rudd AG, Hoffman A, Paley L, Bray B. 20 years of researching stroke through audit. Clin Rehabil. 2018; 32:997-1006.

15. Fisher RJ, Byrne A, Chouliara N, Lewis S, Paley L, Hoffman A, Rudd A, Robinson T, Langhorne P, Walker MF. Effectiveness of Stroke Early Supported Discharge: Analysis from a National Stroke Registry. Circulation: Cardiovascular Quality and Outcomes. 2020; (In press)

16. Fisher R, Chouliara N, Byrne A, Lewis S, Langhorne P, Robinson T, Waring J, Geue C, Hoffman A, Paley L, Rudd A, Walker MF. What is the impact of large-scale implementation of stroke Early Supported Discharge? A mixed methods realist evaluation study protocol. Implement Sci. 2019; 14:61.

17. NHS Midlands and East. NHS Midlands and East Stroke Service Specification. https://www.england.nhs.uk/mids-east/wp-content/uploads/sites/7/2018/03/final-stroke-spec.pdf. 2012; Accessed 15 Mar 2019.

18. Sentinel Stroke National Audit Programme. A Guide to Therapy Data. https://ssnap.zendesk.com/hc/en-us/articles/360001955854-Download-fullpack-of-Therapy-Report. 2017; Accessed 6 Mar 2019.

19. Sentinel Stroke National Audit Programme. National Clinical results. https://www.strokeaudit.org/results/Clinical-audit/National-Results.aspx. 2016; Accessed 6 Mar 2019.

20. Department of Health. Measures from the Adult Social Care Outcomes Framework, England. https://digital.nhs.uk/data-and-information/publications/statistical/adult-social-care-outcomes-framework-ascof. 2015-2016; Accessed 6 Mar 2019.

21. Turner M, Barber M, Dodds H, Murphy D, Dennis M, Langhorne P, Macleod MJ. Implementing a simple care bundle is associated with improved outcomes in a national cohort of patients with ischemic stroke. Stroke. 2015; 46:1065-70.

22. Teale E, Forster A, Munyombwe T, Young JB. A systematic review of case-mix adjustment models for stroke. Clin Rehabil. 2012; 26:771-786.

23. Raine R, Fitzpatrick R, Barratt H, Bevan G, Black N, Boaden R, Bower P, Campbell M, Denis JL, Devers K, Dixon-Woods M, Fallowfield L, Forder J, Foy R, Freemantle N, Fulop NJ, Gibbons E, Gillies C, Goulding L, Grieve R, Grimshaw J, Howarth E, Lilford RJ, McDonald R, Moore G, Moore L, Newhouse R, O'Cathain A, Or Z, Papoutsi C, Prady S, Rycroft-Malone J, Sekhon J, Turner S, Watson SI, Zwarenstein M. Challenges, solutions and future directions in the evaluation of service innovations in health care and public health. Health Serv Deliv Res. 2016; 4:16.

For peer review only - http://bmjopen.bmj.com/site/about/guidelines.xhtml

24. Morris S, Hunter RM, Ramsay AIG, Boaden R, McKevitt C, Perry C, Pursani N, Rudd AG, Schwamm LH, Turner SJ, Tyrrell PJ, Wolfe CDA, Fulop NJ. Impact of centralising acute stroke services in English metropolitan areas on mortality and length of hospital stay: difference-in-differences analysis. BMJ. 2014; 349:g4757.

25. Rafferty A, Walthery P, King-Hele S. Analysing Change Over Time: repeated cross sectional and longitudinal survey data. UK Data Service, University of Essex and University of Manchester. 2015.

26. Propensity Score Matching in Stata using teffects. https://www.ssc.wisc.edu/sscc/pubs/stata_psmatch.htm. 2015; Accessed 28 Nov 2019.

27. Sekhon, J. S. Multivariate and Propensity Score Matching Software with Automated Balance Optimization: The Matching package for R. Journal of Statistical Software. 2011; 42:1-52.

28. Sentinel Stroke National Audit Programme: Cost and Cost-effectiveness analysis. https://www.strokeaudit.org/SupportFiles/Documents/Health-Economics/Health-economic-report-2016.aspx. Accessed 8 Jun 2020.

29. Cobley CS, Fisher RJ, Chouliara N, Kerr M, Walker MF. A qualitative study exploring patients' and carers' experiences of Early Supported Discharge Services after stroke. Clin Rehabil. 2013; 27:750-757.

30. Chouliara N, Fisher RJ, Kerr M, Walker MF. Implementing evidence-based Stroke Early Supported Discharge services: a qualitative study of challenges, facilitators and impact. Clin Rehabil. 2014; 28:370-377.

31. Swinkels A, Mitchell T. Delayed transfer from hospital to community settings: the older person's perspective. Health Soc Care Community. 2008; 17:45-53.

32. Tistad M, von Koch L. Usual Clinical Practice for Early Supported Discharge after Stroke with Continued Rehabilitation at Home: An Observational Comparative Study. PLoS ONE. 2015; 10:e0133536. doi:10.1371/journal.pone.0133536

33. Bråndal1 A, Eriksson M, Glader EL, Wester P. Effect of early supported discharge after stroke on patient reported outcome based on the Swedish Riksstroke registry. BMC Neurology. 2019; 19:40. https://doi.org/10.1186/s12883-019-1268-8

34. Fisher RJ, Gaynor C, Kerr M, Langhorne P, Anderson C, Bautz-Holter E, Indredavik B, Mayo NE, Power M, Rodgers H, Rønning OM, Holmqvist LW, Wolfe CDA, Walker MF. A consensus on stroke: early supported discharge. Stroke. 2011; 42:1392-7.

35. Fisher RJ, Cobley CS, Potgieter I, Moody A, Nouri F, Gaynor C, Byrne A, Walker MF. Is Stroke Early Supported Discharge still effective in practice? A prospective comparative study. Clin Rehabil. 2016; 30:268-276.

36. Gittins M, Lugo-Palacios DG, Paley L, Bray B, Bowen A, Vail A, Gannon B, Tyson S. How do patients pass through stroke services? Identifying stroke care pathways using national audit data. Clin Rehabil. 2020; 34:698-709.

For peer review only

For peer review only - http://bmjopen.bmj.com/site/about/guidelines.xhtml

Figure Legends

Figure 1: Study design flow chart
SSNAP, Sentinel Stroke National Audit Programme; LOS, length of stay; RCS, repeated cross sectional; WISE, What is the Impact of Stroke Early supported discharge?; ESD, early supported discharge.

For peer review only

For peer review only - http://bmjopen.bmj.com/site/about/guidelines.xhtml

Tables

Table 1| Characteristics of 2015-2016 patient cohort

**Patient Characteristics**		**ESD (n = 8648)**	**Non-ESD (n = 22908)**
Age,y	<60	1422 (16.4)	3674 (16.0)
	60-69	1692 (19.6)	3932 (17.2)
	70-79	2646 (30.6)	6080 (26.5)
	80-89	2373 (27.4)	6873 (30.0)
	>89	515 (6.0)	2349 (10.3)
Gender	Female	3779 (43.7)	11123 (48.6)
	Male	4869 (56.3)	11785 (51.4)
Already inpatient at time of stroke	No	8384 (97.0)	21913 (95.7)
	Yes	264 (3.1)	995 (4.3)
Congestive heart failure prior to admission	No	8300 (96.0)	21914 (95.7)
	Yes	348 (4.0)	994 (4.3)
Hypertension prior to admission	No	3905 (45.2)	10609 (46.3)
	Yes	4743 (54.9)	12299 (53.7)
Atrial fibrillation prior to admission	No	7278 (84.2)	18892 (82.5)
	Yes	1370 (15.8)	4016 (17.5)
Diabetes prior to admission	No	6878 (79.5)	18074 (78.9)
	Yes	1770 (20.5)	4834 (21.1)
Stroke/TIA prior to admission	No	6573 (76.0)	16808 (73.4)
	Yes	2075 (24.0)	6100 (26.6)
Modified Rankin Scale score before stroke	0	5688 (65.8)	12796 (55.9)
	>0	2960 (34.2)	10112 (44.1)
NIHSS score on arrival	0	1041 (12.0)	3267 (14.3)
	1-5	4640 (53.7)	11252 (49.1)
	6-14	2253 (26.1)	5596 (24.4)
	15-24	636 (7.4)	2322 (10.1)
	>24	78 (0.9)	471 (2.1)
Type of stroke	Infarction	7804 (90.8)	20772 (91.2)
	Primary Intracerebral Haemorrhage	789 (9.2)	2002 (8.8)
Modified Rankin Scale score at inpatient discharge	0	1046 (12.1)	4016 (17.5)
	1	2259 (26.1)	5024 (21.9)
	2	1928 (22.3)	3420 (14.9)
	3	2189 (25.3)	3838 (16.8)
	4	1118 (12.9)	4154 (18.1)
	5	108 (1.3)	2456 (10.7)
Median (IQR) total length of hospital stay in days		6.9 (2.9-18.8)	6.0 (2.2-24.8)

Values are numbers (percentages) unless stated otherwise. NIHSS indicates National Institutes of Health Stroke Scale; TIA, transient ischemic attack; and IQR, interquartile range.

For peer review only

For peer review only - http://bmjopen.bmj.com/site/about/guidelines.xhtml

Table 2| Association between having received ESD on the care pathway and inpatient length of stay 2015-2016

Inpatient length of stay models	Unadjusted		Adjusted*	
Patients	30975		30791	
Admitting hospitals	55		55	
Patients per admitting hospital: Min	27		26	
Mean	563.2		559.8	
Max	1331		1310	
Intraclass correlation coefficient	0.05		0.08	
**ESD on the care pathway**	**Coefficient (95% CI)**	**P value**	**Coefficient (95% CI)**	**P value**
Received ESD	0.08 (0.04 to 0.12)	< 0.001	0.15 (0.12 to 0.18)	< 0.001

Model coefficients are on the natural log scale; significant results were back-transformed, that is, exponentiated to obtain percentage change in length of hospital stay (reported in text). ESD indicates early supported discharge; NIHSS, National Institutes of Health Stroke Scale; ASCOF, Adult Social Care Outcomes Framework; and SSNAP, Sentinel Stroke National Audit Programme.

** Adjusted for age, sex, prestroke independence, comorbidities, NIHSS score on admission, type of stroke and modified Rankin score at discharge from hospital (patient level); ASCOF rate and hospital SSNAP rating score (admitting hospital level)*

For peer review only

For peer review only - http://bmjopen.bmj.com/site/about/guidelines.xhtml

Table 3| Matched analysis between patients who received ESD on the care pathway and similar patients who did not 2015-2016⁺

**Inpatient matching models**	**Between***		**Within***	
Patients	30791 (8593 ESD + 22198 non-ESD)		10449 (4572 ESD + 5877 non-ESD)	
Admitting hospitals	55		14	
**ESD on the care pathway**	**Coefficient (95% CI)**	**P value**	**Coefficient (95% CI)**	**P value**
Received ESD	0.07 (0.03 to 0.11)	< 0.001	0.08 (0.05 to 0.12)	< 0.001

Model coefficients are on the natural log scale; significant results were back-transformed, that is, exponentiated to obtain percentage difference in length of hospital stay (reported in text). ESD indicates early supported discharge; and NIHSS, National Institutes of Health Stroke Scale.

⁺ This is a sensitivity analysis using propensity score matching to complement the multilevel model results presented in Table 2. Between means patients in all hospitals were matched even if the hospitals did not offer ESD whereas Within means only hospitals with patients who did and did not receive ESD were matched

** Adjusted for age, sex, prestroke independence, comorbidities, NIHSS score on admission, type of stroke and modified Rankin score at discharge from hospital (patient level)*

For peer review only

For peer review only - http://bmjopen.bmj.com/site/about/guidelines.xhtml

Table 4| Patient characteristics from 2013-2014 & 2015-2016 cohorts

Patient Characteristics		ESD (n = 11376)	Non-ESD (n = 39417)
Age,y	<60	1847 (16.2)	6212 (15.8)
	60-69	2239 (19.7)	6647 (16.9)
	70-79	3467 (30.5)	10543 (26.8)
	80-89	3126 (27.5)	12087 (30.7)
	>89	697 (6.1)	3928 (10)
Gender	Female	5024 (44.2)	19458 (49.4)
	Male	6352 (55.8)	19959 (50.6)
Already inpatient at time of stroke	No	11015 (96.8)	37651 (95.5)
	Yes	361 (3.2)	1766 (4.5)
Congestive heart failure prior to admission	No	10895 (95.8)	37859 (96.1)
	Yes	481 (4.2)	1558 (4)
Hypertension prior to admission	No	5115 (45)	18104 (45.9)
	Yes	6261 (55)	21313 (54.1)
Atrial fibrillation prior to admission	No	9536 (83.8)	32118 (81.5)
	Yes	1840 (16.2)	7299 (18.5)
Diabetes prior to admission	No	9031 (79.4)	31343 (79.5)
	Yes	2345 (20.6)	8074 (20.5)
Stroke/TIA prior to admission	No	8636 (75.9)	28935 (73.4)
	Yes	2740 (24.1)	10482 (26.6)
Modified Rankin Scale score before stroke	0	7389 (65)	22847 (58)
	>0	3987 (35.1)	16570 (42)
NIHSS score on arrival	0	1430 (12.6)	6884 (17.5)
	1-5	6020 (52.9)	18802 (47.7)
	6-14	2955 (26)	9162 (23.2)
	15-24	861 (7.6)	3833 (9.7)
	>24	110 (1)	736 (1.9)
Type of stroke	Infarction	10315 (91.2)	35989 (92.1)
	Primary Intracerebral Haemorrhage	998 (8.8)	3079 (7.9)
Modified Rankin Scale score at inpatient discharge	0	1440 (12.7)	8474 (21.5)
	1	2928 (25.7)	8649 (21.9)
	2	2562 (22.5)	5456 (13.8)
	3	2817 (24.8)	6090 (15.5)
	4	1471 (12.9)	6733 (17.1)
	5	158 (1.4)	4015 (10.2)
Time period	2013-2014	3065 (26.9)	21841 (55.4)
	2015-2016	8311 (73.1)	17576 (44.6)
Median (IQR) total length of hospital stay in days			
	2013-2014	7.7 (3.2-23.8)	6.3 (2.5-22.1)
	2015-2016	6.8 (2.9-18.5)	5.7 (2.1-24.6)

Values are numbers (percentages) unless stated otherwise. NIHSS indicates National Institutes of Health Stroke Scale; TIA, transient ischemic attack; and IQR, interquartile range.

For peer review only

For peer review only - http://bmjopen.bmj.com/site/about/guidelines.xhtml

Table 5| Association between having received ESD on the care pathway and inpatient length of stay 2013-2016

**Inpatient length of stay models**	**Unadjusted**		**Adjusted***	
Patients	49667		49266	
Admitting hospitals	41		41	
Patients per admitting hospital: Min	115		106	
Mean	1211.4		1201.6	
Max	2512		2446	
Intraclass correlation coefficient	0.04		0.08	
**ESD on the care pathway**	**Coefficient (95% CI)**	**P value**	**Coefficient (95% CI)**	**P value**
Received ESD	0.14 (0.09 to 0.20)	< 0.001	0.17 (0.13 to 0.22)	< 0.001
2015-2016 time period	-0.06 (-0.09 to -0.04)	< 0.001	-0.15 (-0.17 to -0.13)	< 0.001
Received ESD * 2015-2016 time period	-0.05 (-0.11 to 0.02)	0.162	0.01 (-0.04 to 0.06)	0.790

Model coefficients are on the natural log scale; significant results were back-transformed, that is, exponentiated to obtain percentage change in length of hospital stay (reported in text). ESD indicates early supported discharge; NIHSS, National Institutes of Health Stroke Scale; ASCOF, Adult Social Care Outcomes Framework; and SSNAP, Sentinel Stroke National Audit Programme.

** Adjusted for age, sex, prestroke independence, comorbidities, NIHSS score on admission, type of stroke and modified Rankin score at discharge from hospital (patient level); ASCOF rate and hospital SSNAP rating score (admitting hospital level)*

For peer review only

For peer review only - http://bmjopen.bmj.com/site/about/guidelines.xhtml

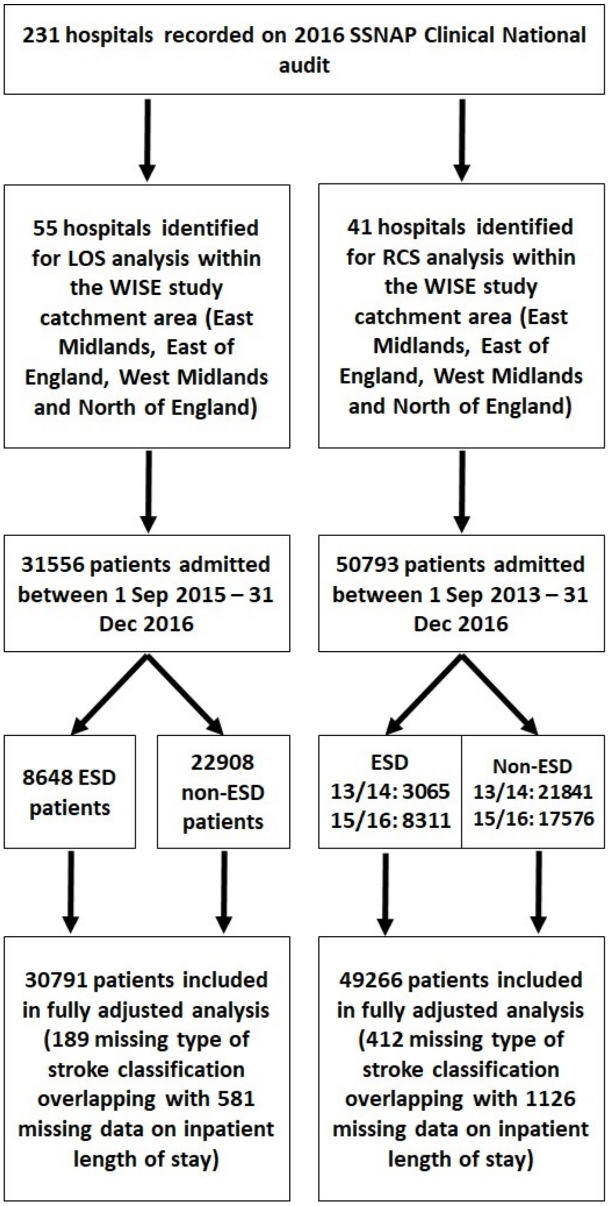

Figure 1: Study design flow chart
SSNAP, Sentinel Stroke National Audit Programme; LOS, length of stay; RCS, repeated cross sectional; WISE, What is the Impact of Stroke Early supported discharge?; ESD, early supported discharge.

138x273mm (96 x 96 DPI)

STROBE Statement—Checklist of items that should be included in reports of ***cross-sectional studies***

	Item No	Recommendation	Page No
**Title and abstract**	1	(*a*) Indicate the study's design with a commonly used term in the title or the abstract	0,1
		(*b*) Provide in the abstract an informative and balanced summary of what was done and what was found	1
**Introduction**			
Background/rationale	2	Explain the scientific background and rationale for the investigation being reported	3
Objectives	3	State specific objectives, including any prespecified hypotheses	4
**Methods**			
Study design	4	Present key elements of study design early in the paper	4,5
Setting	5	Describe the setting, locations, and relevant dates, including periods of recruitment, exposure, follow-up, and data collection	5
Participants	6	(*a*) Give the eligibility criteria, and the sources and methods of selection of participants	5
Variables	7	Clearly define all outcomes, exposures, predictors, potential confounders, and effect modifiers. Give diagnostic criteria, if applicable	6,7
Data sources/ measurement	8*	For each variable of interest, give sources of data and details of methods of assessment (measurement). Describe comparability of assessment methods if there is more than one group	5
Bias	9	Describe any efforts to address potential sources of bias	8
Study size	10	Explain how the study size was arrived at	4
Quantitative variables	11	Explain how quantitative variables were handled in the analyses. If applicable, describe which groupings were chosen and why	6,7
Statistical methods	12	(*a*) Describe all statistical methods, including those used to control for confounding	7
		(*b*) Describe any methods used to examine subgroups and interactions	8
		(*c*) Explain how missing data were addressed	8
		(*d*) If applicable, describe analytical methods taking account of sampling strategy	7
		(*e*) Describe any sensitivity analyses	9
**Results**			
Participants	13*	(a) Report numbers of individuals at each stage of study—eg numbers potentially eligible, examined for eligibility, confirmed eligible, included in the study, completing follow-up, and analysed	10,11
		(b) Give reasons for non-participation at each stage	N/A
		(c) Consider use of a flow diagram	4
Descriptive data	14*	(a) Give characteristics of study participants (eg demographic, clinical, social) and information on exposures and potential confounders	10,11
		(b) Indicate number of participants with missing data for each variable of interest	N/A
Outcome data	15*	Report numbers of outcome events or summary measures	10,11
Main results	16	(*a*) Give unadjusted estimates and, if applicable, confounder-adjusted estimates and their precision (eg, 95% confidence interval). Make clear which confounders were adjusted for and why they were included	21,24

For peer review only - http://bmjopen.bmj.com/site/about/guidelines.xhtml

		(*b*) Report category boundaries when continuous variables were categorized	20,23
		(*c*) If relevant, consider translating estimates of relative risk into absolute risk for a meaningful time period	N/A
Other analyses	17	Report other analyses done—eg analyses of subgroups and interactions, and sensitivity analyses	10,22
**Discussion**			
Key results	18	Summarise key results with reference to study objectives	12,13
Limitations	19	Discuss limitations of the study, taking into account sources of potential bias or imprecision. Discuss both direction and magnitude of any potential bias	13,14
Interpretation	20	Give a cautious overall interpretation of results considering objectives, limitations, multiplicity of analyses, results from similar studies, and other relevant evidence	14
Generalisability	21	Discuss the generalisability (external validity) of the study results	N/A
**Other information**			
Funding	22	Give the source of funding and the role of the funders for the present study and, if applicable, for the original study on which the present article is based	15

*Give information separately for exposed and unexposed groups.

Note: An Explanation and Elaboration article discusses each checklist item and gives methodological background and published examples of transparent reporting. The STROBE checklist is best used in conjunction with this article (freely available on the Web sites of PLoS Medicine at http://www.plosmedicine.org/, Annals of Internal Medicine at http://www.annals.org/, and Epidemiology at http://www.epidem.com/). Information on the STROBE Initiative is available at www.strobe-statement.org.

For peer review only

For peer review only - http://bmjopen.bmj.com/site/about/guidelines.xhtml

Effect of Stroke Early Supported Discharge on length of hospital stay: Analysis from a National Stroke Registry

Journal:	*BMJ Open*
Manuscript ID	bmjopen-2020-043480.R1
Article Type:	Original research
Date Submitted by the Author:	15-Dec-2020
Complete List of Authors:	Fisher, Rebecca; University of Nottingham<br>Byrne, Adrian; University of Nottingham, Division of Rehabilitation, Ageing and Wellbeing<br>Chouliara, Niki; University of Nottingham<br>Lewis, Sarah; University of Nottingham<br>Paley, Lizz; King's College London<br>Hoffman, Alex; King's College London<br>Rudd, Anthony; King's College London<br>Robinson, Thompson; University of Leicester<br>Langhorne, Peter; University of Glasgow<br>Walker, Marion; University of Nottingham
**Primary Subject Heading**:	Health services research
Secondary Subject Heading:	Rehabilitation medicine
Keywords:	STROKE MEDICINE, REHABILITATION MEDICINE, Health policy < HEALTH SERVICES ADMINISTRATION & MANAGEMENT, Stroke < NEUROLOGY

SCHOLARONE™
Manuscripts

I, the Submitting Author has the right to grant and does grant on behalf of all authors of the Work (as defined in the below author licence), an exclusive licence and/or a non-exclusive licence for contributions from authors who are: i) UK Crown employees; ii) where BMJ has agreed a CC-BY licence shall apply, and/or iii) in accordance with the terms applicable for US Federal Government officers or employees acting as part of their official duties; on a worldwide, perpetual, irrevocable, royalty-free basis to BMJ Publishing Group Ltd ("BMJ") its licensees and where the relevant Journal is co-owned by BMJ to the co-owners of the Journal, to publish the Work in this journal and any other BMJ products and to exploit all rights, as set out in our licence.

The Submitting Author accepts and understands that any supply made under these terms is made by BMJ to the Submitting Author unless you are acting as an employee on behalf of your employer or a postgraduate student of an affiliated institution which is paying any applicable article publishing charge ("APC") for Open Access articles. Where the Submitting Author wishes to make the Work available on an Open Access basis (and intends to pay the relevant APC), the terms of reuse of such Open Access shall be governed by a Creative Commons licence – details of these licences and which Creative Commons licence will apply to this Work are set out in our licence referred to above.

Other than as permitted in any relevant BMJ Author's Self Archiving Policies, I confirm this Work has not been accepted for publication elsewhere, is not being considered for publication elsewhere and does not duplicate material already published. I confirm all authors consent to publication of this Work and authorise the granting of this licence.

For peer review only

Effect of Stroke Early Supported Discharge on length of hospital stay: Analysis from a National Stroke Registry

First author surname: Fisher

Short title: **Effect of Stroke Early Supported Discharge on length of hospital stay**

Rebecca J Fisher, PhD; Adrian Byrne, PhD; Niki Chouliara, PhD; Sarah Lewis, PhD; Lizz Paley, MSc; Alex Hoffman, MSc; Anthony Rudd, MD; Thompson Robinson, MD; Peter Langhorne, PhD; Marion F Walker, PhD.

University of Nottingham, Nottingham, United Kingdom (RF,AB,NC,SL,MW)
King's College London, London, United Kingdom (LP,AH,AR)
University of Leicester, Leicester, United Kingdom (TR)
University of Glasgow, Glasgow, United Kingdom (PL)

Corresponding author

Dr Rebecca Fisher
Division of Rehabilitation, Ageing & Wellbeing
School of Medicine
University of Nottingham
NG7 2UH

+44 (0) 115 82 30253
rebecca.fisher@nottingham.ac.uk

I, the Submitting Author has the right to grant and does grant on behalf of all authors of the Work (as defined in the below author licence), an exclusive licence and/or a non-exclusive licence for contributions from authors who are: i) UK Crown employees; ii) where BMJ has agreed a CC-BY licence shall apply, and/or iii) in accordance with the terms applicable for US Federal Government officers or employees acting as part of their official duties; on a worldwide, perpetual, irrevocable, royalty-free basis to BMJ Publishing Group Ltd ("BMJ") its licensees and where the relevant Journal is co-owned by BMJ to the co-owners of the Journal, to publish the Work in BMJ Open and any other BMJ products and to exploit all rights, as set out in our licence.

The Submitting Author accepts and understands that any supply made under these terms is made by BMJ to the Submitting Author unless you are acting as an employee on behalf of your employer or a postgraduate student of an affiliated institution which is paying any applicable article publishing charge ("APC") for Open Access articles. Where the Submitting Author wishes to make the Work available on an Open Access basis (and intends to pay the relevant APC), the terms of reuse of such Open Access shall be governed by a Creative Commons licence – details of these licences and which Creative Commons licence will apply to this Work are set out in our licence referred to above.

Key words: early supported discharge, stroke delivery, health services research, patient discharge, rehabilitation, evaluation, health policy

Word count: 3,514

Total number of tables and figures: Tables 5; Figures 1.

For peer review only - http://bmjopen.bmj.com/site/about/guidelines.xhtml

For peer review only

Abstract

Objective − The first observational study to investigate the impact of Early Supported Discharge (ESD) on length of hospital stay in real world conditions.

Design − Using historical prospective Sentinel Stroke National Audit Programme (SSNAP) data (1 Jan 2013 – 31 Dec 2016) and multilevel modelling, cross-sectional (2015-2016; 30,791 patients nested within 55 hospitals) and repeated cross-sectional (2013-2014 vs 2015-2016; 49,266 patients nested within 41 hospitals) analyses were undertaken.

Setting − Hospitals were sampled across a large geographical area of England covering the West and East Midlands, the East of England and the North of England.

Participants – Stroke patients whose data were entered into the SSNAP database by hospital teams.

Interventions − Receiving ESD along the patient care pathway

Primary and secondary outcome measures – Length of hospital stay

Results − When adjusted for important case-mix variables, patients who received ESD on their stroke care pathway spent longer in hospital, compared to those who did not receive ESD. The percentage increase was 15.8% (95% CI 12.3% - 19.4%) for the 2015-2016 cross-sectional analysis and 18.8% (95% CI 13.9% - 24.0%) for the 2013-2014 versus 2015-2016 repeated cross-sectional analysis. On average, the increased length of hospital stay was approximately 1 day.

Conclusions − This study has shown that by comparing ESD and non-ESD patient groups matched for important patient characteristics, receiving ESD resulted in a 1 day increase in length of hospital stay. The large reduction in length of hospital stay overall, since original trials were conducted, may explain why a reduction was not observed. The longer term benefits of accessing ESD need to be investigated further.

Study registration − http://www.isrctn.com/ISRCTN15568163

For peer review only - http://bmjopen.bmj.com/site/about/guidelines.xhtml

Strengths and limitations of this study

- First observational study comparing length of hospital stay in real world settings for patients accessing Early Supported Discharge (ESD) compared to those that did not
- Use of datasets from the Sentinel Stroke National Audit Programme (SSNAP), permitting sample sizes of 30,791 and 49,266 patients
- Accounts for the variation that exists between patients clustered within their admitting hospitals by employing a multilevel modelling approach and reinforces group comparisons using a propensity score matching process
- Unable to account for residual confounding
- Did not investigate the longer term benefits of accessing ESD

For peer review only

For peer review only - http://bmjopen.bmj.com/site/about/guidelines.xhtml

Introduction

Stroke is one of the main causes of adult disability and there is strong research evidence that provision of stroke specialist rehabilitation enhances recovery.[1] Stroke Early Supported Discharge (ESD) is a multidisciplinary team intervention aimed at facilitating discharge from hospital and providing stroke specialist rehabilitation at home.[2] Based on cumulative evidence from clinical trials, stroke care guidelines in the UK and worldwide, recommend the provision of ESD as part of an evidence-based stroke care pathway.[3-8]

In many high-income countries, ESD has not been well developed in practice with a lack of large scale implementation.[9] In contrast, in the UK, considerable efforts have been made to implement ESD, although types of service differ across the country, and in some regions, ESD is still not offered at all.[10] Where ESD has been delivered, it is unclear whether benefits of the ESD intervention, as suggested in clinical trials, are achieved in practice.

One of the attractive features of the ESD intervention as reported in clinical trials, has been reduction in length of hospital stay, with the most recent Cochrane systematic review suggesting a reduction of 6 days.[2] This reduction has contributed to the reported cost-effectiveness of combined stroke unit care and ESD.[11] Small scale evaluation of ESD operating in localised areas have suggested smaller, but significant, reductions in length of acute hospital stay, but it remains unknown what impact ESD is having over a larger scale.[12] Average length of hospital stay for all stroke patients discharged alive fell from 40 days in 2001 to 20 days in 2013 and has remained around this level since then.[13] This trend over time has meant more stroke unit beds are available for new patients but it has also increased demand for patient care in the community post hospital discharge.

The Sentinel Stroke National Audit programme (SSNAP) is the national stroke register of England, Wales and Northern Ireland in which all acute admitting hospitals and post-acute

For peer review only - http://bmjopen.bmj.com/site/about/guidelines.xhtml

stroke teams are mandated to participate.[14] SSNAP has played a key role in monitoring performance and improving provision of acute stroke care. Collection of SSNAP data across the stroke care pathway now offers a unique opportunity to investigate the large scale impact of post-acute interventions such as ESD. The study reported here, and in a parallel paper exploring the effectiveness of ESD service models, investigates if trial-based benefits of ESD are realised in practice, with the aim to inform evidence based improvements.[15] Here, we focus on the impact of ESD on length of hospital stay.

Methods

Study design

We present results from an observational cohort study (Figure 1), conducted as part of an overall mixed method study.[16] The study protocol was approved by a University of Nottingham Ethics Committee and the UK Healthcare Quality Improvement Partnership Data Access Request Group. Data access requests should be directed to Healthcare Quality Improvement Partnership as the data controller and SSNAP as the data provider. The study protocol including statistical analysis plan is available online.[16] We determined a priori, a sample size of 21,760 patients for a study power of 80% to detect standardised effect sizes of 0.25 for each outcome.

In this paper, we have used two different study designs: (1) a cross-sectional analysis (SSNAP data from 2015-2016 time period) comparing length of hospital stay between patients who did and did not have ESD on their care pathway, with adjustment for hospital and individual confounding factors; (2) repeated cross-sectional analysis using two sets of

For peer review only - http://bmjopen.bmj.com/site/about/guidelines.xhtml

SSNAP data (2013-2014 and 2015-2016) to establish whether length of hospital stay changed over time and whether any change was attributable to ESD.

Setting

Hospitals (and associated ESD services) were sampled across a large geographical area of England. The sampling strategy was devised in accordance with the overall mixed method study design and included all ESD services in specific regions of England.[16] Here we report findings from the quantitative investigation of ESD effect on length of hospital stay across West and East Midlands and East of England, (across which a specific initiative to promote ESD was initiated in 2010) and the North of England, a region with a defined lack of ESD.[10,17] We have included all main referring hospitals within the defined geographical area as well as referring hospitals whose patients did (or did not) go onto receive ESD along their care pathway within the catchment area.

Data sources & participants

Patient-level SSNAP data are entered by clinical teams onto a secure webtool with real time data validations to ensure data quality.[14] Historical prospective clinical (patient-level) SSNAP data were obtained from the SSNAP team with permission from Healthcare Quality Improvement Partnership. The first study design involved 55 admitting hospitals and SSNAP data for all patients admitted during 1 Sep 2015 – 31 Dec 2016 (31,556 stroke patients). The second study design included 41 admitting hospitals, which were those of the 55 hospitals for which we could obtain SSNAP data for all patients admitted during two time periods, 1 Sep 2013 – 31 Dec 2014 and 1 Sep 2015 – 31 Dec 2016 (50,793 stroke patients).

Outcomes

For peer review only - http://bmjopen.bmj.com/site/about/guidelines.xhtml

Length of hospital stay was defined as the total length of hospital stay per patient (as an inpatient) from arrival at the admitting hospital (or time of stroke onset, if already an inpatient), to the time of discharge from the last inpatient setting, including time spent at any other inpatient setting. Owing to the positive skewness of the underlying distribution, length of hospital stay was natural log transformed before undertaking the statistical modelling and all effect sizes were exponentiated afterwards to aid interpretation.

Confounding factors

In order to investigate the effect of ESD on length of hospital stay, we identified a need to control for the overall standard of hospital care and also the influence of provision of social care. At the hospital level we included two confounding variables: a hospital SSNAP rating score and a measure of delayed transfers of care from hospital, derived from the Adult Social Care Outcomes Framework (ASCOF).

The hospital rating scores used in this study were an overall quality rating for each hospital obtained from SSNAP (total key indicator score derived across ten domains of stroke care with adjustments made for case ascertainment levels and the quality of data submitted to SSNAP).[18] SSNAP produced performance rating scores for admitting hospitals during the periods of interest and we used the score produced during the latter part of the period of interest for each analysis.[19] This score for each admitting hospital was used as an indication of the overall standard of inpatient care after the onset of stroke. The ASCOF data reports the average daily rate of delayed transfers of care per 100,000 population aged 18 and over at local authority level. Data were aggregated up to National Health Service Trust level, using averages where multiple local authorities were associated with one National Health Service Trust. Again we focused on the scores produced during the latter part of the period of interest in line with the SSNAP hospital performance ratings.[20]

For peer review only - http://bmjopen.bmj.com/site/about/guidelines.xhtml

In order to account for comparison between different groups of individual patients, we also included variables at the patient level. These were stroke patient characteristics, reflecting validated stroke case-mix models and collected as part of the SSNAP data set. These were age at admission, sex, pre-stroke independence, cardiovascular comorbidities, NIH stroke scale score on admission, type of stroke and modified Rankin score at discharge from hospital.[21,22]

Statistical analyses

We employed a linear multilevel model framework for both study designs whereby patients (level 1) were clustered within admitting hospitals (level 2) in an approach consistent with previous observational studies of this type.[15,21-24] Within our multilevel models, we adjusted for a range of patient and hospital characteristics as covariates. We chose multilevel modelling to evaluate the effect of ESD on length of hospital stay as it could accommodate and appreciate the variation that existed within and between different admitting hospitals. Furthermore, the intraclass correlation coefficient was calculated as a measure of proportion of the total variance in length of hospital stay, which was attributable to variance within admitting hospitals as opposed to between admitting hospitals.

The adequacy of different statistical models was compared using the log-likelihood, Akaike Information Criterion and Bayesian Information Criterion values from single level and multilevel regression models with multilevel preferable on each occasion. Multicollinearity was investigated by examining variance inflation factor scores of all predictor variable sets and was found not to be an issue. Covariate linearity was examined by checking the consistency of a linear trend in relation to each outcome variable. Regarding the impact of missing data, we assessed the mean change in the outcome variable between the ESD and non-ESD groups in relation to missing predictor data (which amounted to a loss of 0.6% of

For peer review only - http://bmjopen.bmj.com/site/about/guidelines.xhtml

the total sample size); no substantial differences were found as we observed a mean difference of less than 0.3%.

Analysis of cross-sectional data from 2015-2016

A quasi-experimental cross-sectional design was used in which patients who received ESD on their care pathway were compared with patients that had not (non-ESD). Hence across 55 admitting hospitals covering the 1 Sep 2015 – 31 Dec 2016 data period, we investigated the effect of ESD being on the care pathway (yes/no) for stroke patients on their total length of hospital stay from admitting hospital until being discharged from the last inpatient setting.

Analysis of repeated cross-sectional data from 2013-2014 and 2015-2016

This analysis was to establish whether length of hospital stay changed over time i.e. between the earlier (1 Sep 2013 – 31 Dec 2014) and later time periods (1 Sep 2015 – 31 Dec 2016), and whether any change was attributable to ESD, as ESD provision was higher in the later time period.[10] Patients formed distinct groups over the two time periods as they were either admitted in the 2013-2014 time period or the 2015-2016 time period with no patient covering both time periods.[25] The same multilevel model approach was used as in the cross-sectional analysis, but also incorporating an effect of time (13/14 or 15/16), and an interaction between time and the effect of ESD which modelled whether the effect of ESD was similar in the two time periods. The main effect of receiving ESD (with adjustment for time) indicated the effect of ESD on length of hospital stay in the first time period. An interaction between time and ESD would indicate whether there was any difference in the effect of ESD on length of hospital stay between the first and second time periods.

Sensitivity analysis using propensity score matching

For peer review only - http://bmjopen.bmj.com/site/about/guidelines.xhtml

Since it was important to ensure that the effects of ESD were not the result of potential confounding factors, we also used an alternative method of controlling for confounders, i.e. propensity score matching, as a sensitivity analysis. Propensity score matching involved matching patients with ESD on their pathway with similar patients who did not, based on their patient characteristics. In this case, we explored two alternative approaches to propensity score matching, firstly matching patients with ESD to patients without ESD in the same hospital, i.e. within-admitting hospital matching.[26] This resulted in a truncated sample size as only hospitals with both ESD and non-ESD patients could contribute to this first matching analysis.

Secondly, we then allowed the matching to occur with similar patients without ESD in different hospitals, i.e. between-admitting hospital matching.[27] This second approach enabled us to use a larger sample size as all hospitals could contribute to the probability estimate of whether a patient received ESD or not regardless of what their patients actually received. In order to ensure greater accuracy in the latter analysis, we used a stratified matching process so that patients were only matched to patients in a hospital with a similar proportion of patients having access to ESD.

A two tailed significance level of 0.05 was used in all hypothesis tests. We carried out the majority of analyses using Stata/SE 15.1 (Statacorp) and the R package Matching was used to undertake the between-admitting hospital propensity score matching.

Patient and Public Involvement

The design and conduct of this study was informed through discussion with the Nottingham Stroke Partnership group. Two stroke survivor members were part of the study steering group and advised on a lay summary of our study findings.

Results

For peer review only - http://bmjopen.bmj.com/site/about/guidelines.xhtml

Analysis of cross-sectional data from 2015-2016

Table 1 presents descriptive statistics of the patient and admitting hospital level variables and the median number of days spent as an inpatient disaggregated by patients who received ESD (ESD; n=8648) or not (non-ESD; n=22908) on the care pathway. Compared to patients who did not receive ESD, those who did were younger and more likely to be independent (lower pre-morbid modified Rankin Scale score) before stroke as well as more independent at inpatient discharge (lower post-morbid modified Rankin Scale score). ESD patients were also more likely to have been admitted to a hospital with a higher SSNAP score rating and higher rate of delayed transfers.

Before adjustment, ESD patients had a longer length of hospital stay than non-ESD patients; median 6.9 (interquartile range: 2.9-18.8) compared to 6.0 days (interquartile range: 2.2-24.8). Following multilevel model analysis, after controlling for patient and hospital level characteristics, the mean length of stay for patients who received ESD along their care pathway compared to those who did not remained significantly longer by 15.8% (95% CI 12.3% - 19.4%) (Table 2). Similarly, from the sensitivity analysis using propensity score matching, patients who received ESD on their care pathway, on average, stayed in hospital longer (when controlling for all other patient characteristics, and hospital SSNAP and ASCOF scores) (Table 3). The within-admitting hospital propensity score matching process (n=10449) suggested ESD patients stayed in hospital longer by an average of 8.7% (95% CI 4.8% – 12.6%). The between-admitting hospital propensity score matching process (n=30791) found that ESD patients stayed in hospital longer by an average of 7.3% (95% CI 3.0% – 11.8%).

Analysis of repeated cross-sectional data from 2013-2014 and 2015-2016

For peer review only - http://bmjopen.bmj.com/site/about/guidelines.xhtml

Table 4 presents descriptive statistics of the patient and admitting hospital level variables used in the repeated cross-sectional analysis, as well as the median number of days spent as an inpatient disaggregated by patients who received ESD (n=11376) or not (non-ESD; n=39417) on the care pathway across the two time periods. This descriptive repeated cross-sectional analysis showed that the numbers of patients who received ESD significantly increased over the two time periods. Moreover, across the two time periods, ESD patients were younger, more likely to be male and more likely to have pre-morbid independence, as evidenced by a lower modified Rankin Scale score. Before adjustment, ESD patients were recorded as having a longer length of stay in hospital than non-ESD patients; median 7.7 (interquartile range: 3.2-23.8) compared to 6.3 days (interquartile range: 2.5-22.1) in 2013-2014, and 6.8 (interquartile range: 2.9-18.5) compared to 5.7 days (interquartile range: 2.1-24.6) in 2015-2016.

Table 5 presents the results from the linear multilevel model. Again, we controlled for patient characteristics, SSNAP admitting hospital score and average ASCOF rates for each admitting hospital. There was no significant interaction between receiving ESD and time period (p = 0.79), so that the effect of ESD was similar in both time periods, despite more patients accessing ESD over time. Therefore, the main effect of ESD across both time periods was estimated to increase length of hospital stay by an average of 18.8% (95% CI 13.9% - 24.0%) compared to patients who did not receive ESD on their care pathway. Furthermore, the main effect of the 2015-2016 time period compared to the 2013-2014 time period was found to significantly reduce the length of hospital stay by an average of 14.0% (95% CI 12.2% - 15.9%).

The degree of clustering was low in both adjusted multilevel models (adjusted intraclass correlation coefficients respectively, 0.08, 0.08) implying that the total variance we observed

For peer review only - http://bmjopen.bmj.com/site/about/guidelines.xhtml

in the length of hospital stay data was more greatly accounted for by the patients than by the admitting hospitals.

Discussion

This study has investigated the effect of ESD on length of hospital stay in real world settings, with the aim to inform evidence based improvements in stroke care. Unadjusted findings indicated that stroke patients who received ESD spent approximately one day longer in hospital than all other patients, however, those who received ESD differed in characteristics from those who did not. With adjustment using two different designs and alternative analyses for the differing case-mix, length of hospital stay remained longer for those who received ESD on their care pathway. This contrasts with findings from original ESD randomised controlled trials, in which a reduction in length of hospital stay was reported.[2]

This is an important finding, particularly when considering implementation of ESD in practice. The promise of reducing length of hospital stay made ESD attractive with regard to cost savings and is likely to have contributed to widespread implementation in England.[10,11] The economic implications of ESD in the real world have previously been investigated through simulation modelling (informed by SSNAP data similar to data used here).[28] The authors reported an estimated mean length of hospital stay for ESD patients that was 8 days less than non-ESD patients and thereby derived cost savings associated with modelling increased use of ESD.

However, an important difference with that economic analysis and the study reported here relates to the fact that we used actual and total inpatient length of stay data combining different inpatient settings where applicable on a per patient basis. Analysis showed that ESD patients were younger and more likely to have a lower modified Rankin score at inpatient

For peer review only - http://bmjopen.bmj.com/site/about/guidelines.xhtml

discharge. The simulation modelling findings drew upon sample statistics derived from patient distributions for each separate inpatient setting stratified by age category and modified Rankin Scale score and their results were driven by assuming more severe stroke survivors received ESD. What our study adds is that when comparing ESD and non-ESD patient groups matched for important patient characteristics, receiving ESD does not relate to a reduction in length of hospital stay.

Another important consideration is that average length of hospital stay for stroke survivors overall has reduced dramatically over the last twenty years, the latter decade of which our study captures between the two cohorts in this paper (i.e. 2013-2014 and 2015-2016).[13] This makes it less likely for an intervention like ESD to have an effect (in comparison to the years 1997-2004 when the original UK clinical trials were conducted).[2] That ESD in this study was associated with an average increase in length of stay of 1 day, could be interpreted as the need for additional time to facilitate the transfer of care to the ESD team, rather than stroke survivors simply leaving hospital without support (if ESD was not available). Previous studies have also highlighted transfer problems relating to lack of joint working between health and social care.[29-31] Our parallel paper added to this debate by highlighting the importance of access to a social worker as part of the ESD team.[15]

What was not possible to investigate in this study was whether access to ESD (despite an additional day in hospital) was associated with improved patient outcomes over the longer term. Recent observational studies in Sweden suggest patient and care-giver benefits related to provision of ESD in regular clinical practice, in line with our previous study findings from England.[32,33] We suggest routine collection of additional validated patient outcome measures (e.g. measuring activities of daily living, general health/mood and quality of life) at longer follow-up periods in national stroke audits or registries is required.[2,34,35]

For peer review only

For peer review only - http://bmjopen.bmj.com/site/about/guidelines.xhtml

Like other observational studies, this study had its limitations. Although numbers of patients were sufficient for the analysis undertaken, by focusing on specific regions of England, transferability of findings could be questioned, particularly outside England. Other limitations include potential sources of residual confounding and also reliance on SSNAP data being accurately reported by hospital teams. Length of hospital stay data is inherently variable and whilst we tried to include a proxy measure for other sources of delays in discharge, analysis would have benefitted from social care related patient level variables.[28,32] Patients also experienced a series of hospital stays (e.g. transfer between acute and rehabilitation wards), relying on accurate transfer of SSNAP records between hospitals and resulting in us focusing on total length of hospital stay and ESD provision on a care pathway.[36] What this means is that the impact of ESD on a particular hospital may not be apparent, which may be of more interest to providers of hospital services.

Conclusion

Original clinical trials of ESD were conducted across the world and implementation of ESD is recommended in many countries' stroke guidelines.[4-9] This study investigated the impact of ESD in real world settings and focused on length of hospital stay. ESD was not associated with a reduction in length of hospital stay as previously reported in clinical trials, although the increased average length of stay was just 1 day. This highlights the importance of investigating whether trial-based outcomes of interventions are realised in the real world. What remains to be investigated and reported are the benefits of accessing ESD over the longer term. Also by focusing analysis on patients who would have been eligible, but did not receive ESD, we have highlighted an important gap in service provision.

For peer review only - http://bmjopen.bmj.com/site/about/guidelines.xhtml

Contributorship Statement

All named authors made a substantial contribution across a number of areas including study design, data analysis, interpretation of findings, drafting/revising the manuscript and approving the final version for publication. All authors agree to be accountable for respective aspects of the work ensuring that questions related to the accuracy or integrity of any part of the work are appropriately investigated and resolved.

RF (Principal Investigator) led in the original design of the study protocol, with input from MW, SL, TR, PL, AB and NC. LP, AH and AR are Sentinel Stroke National Audit Programme (SSNAP) collaborators and advised on access to audit data and appropriate use for research purposes.

RF led running of the study, with AB, SL leading on statistical methodology, obtaining data from SSNAP, data analysis and interpretation, and article writing and approval; NC, MW, TR, PL advised RF on overall study design, data interpretation, article writing and approval; LP, AH, AR are Sentinel Stroke National Audit Programme (SSNAP) collaborators and were involved in data sharing agreements, advice on data handling, analysis, article writing and approval.

Acknowledgments

We thank the many people and organizations participating in SSNAP and members of the SSNAP collaboration. We also thank Trevor Gard and Frances Cameron, stroke survivors who were members of our study steering group.

Sources of Funding

This research was funded by the National Institute for Health Research (NIHR), Health Services and Delivery Research (HS&DR) Programme (16/01/17). RF is funded by the

For peer review only

Stroke Association (TSA LECT 2016/01 Stroke Association HRH the Princess Margaret Senior Lecturer Award). TR is an NIHR Senior Investigator. The Sentinel Stroke National Audit Programme is commissioned by the Healthcare Quality Improvement Partnership and funded by National Health Service (NHS) England and the Welsh Government.

Disclosures

The views expressed are those of the authors and not necessarily those of the NHS, the NIHR or the Department of Health and Social Care.

Competing interests

All authors declare they have no competing interests.

Patient consent

Not required.

Data sharing agreement

Data access requests should be directed to Healthcare Quality Improvement Partnership as the data controller and SSNAP as the data provider.

References

1. Langhorne P, Bernhardt J, Kwakkel G. Stroke rehabilitation. The Lancet. 2011; 377:1693–702.

2. Langhorne P, Baylan S. Early Supported Discharge Trialists. Early supported discharge services for people with acute stroke. Cochrane Database Syst Rev. 2017; 7:CD000443.

3. Intercollegiate Stroke Working Party. National clinical guideline for stroke. 5th ed. London: Royal College of Physicians. 2016.

4. The National Institute for Health and Care Excellence. Stroke rehabilitation in adults. NICE guidelines (clinical guideline CG 162). 2013.

5. The European Stroke Organization (ESO) Executive Committee and the ESO Writing Committee. Guidelines for Management of Ischaemic Stroke and Transient Ischaemic Attack 2008. http://www.congrex-switzerland.com/fileadmin/files/2013/eso-stroke/pdf/ESO08_Guidelines_Original_english.pdf. 2008; Accessed 28 Aug 2019.

For peer review only - http://bmjopen.bmj.com/site/about/guidelines.xhtml

For peer review only

6. Adeoye O, Nyström KV, Yavagal DR, Luciano J, Nogueira RG, Zorowitz RD, Khalessi AA, Bushnell C, Barsan WG, Panagos P, Alberts MJ, Tiner AC, Schwamm LH, Jauch EC. Recommendations for the Establishment of Stroke Systems of Care: A 2019 Update. A policy statement from the American Stroke Association. Stroke. 2019; 50:e187-e210.

7. Hebert D, Lindsay MP, McIntyre A, Kirton A, Rumney PG, Bagg S, Bayley M, Dowlatshahi D, Dukelow S, Garnhum M, Glasser E, Halabi ML, Kang E, MacKay-Lyons M, Martino R, Rochette A, Rowe S, Salbach N, Semenko B, Stack B, Swinton L, Weber V, Mayer M, Verrilli S, DeVeber G, Andersen J, Barlow K, Cassidy C, Dilenge ME, Fehlings D, Hung R, Iruthayarajah J, Lenz L, Majnemer A, Purtzki J, Rafay M, Sonnenberg LK, Townley A, Janzen S, Foley N, Teasell R. Canadian stroke best practice recommendations: Stroke rehabilitation practice guidelines, update 2015. Int J Stroke. 2016; 11:459-484.

8. Stroke Foundation. Clinical Guidelines for Stroke Management. Melbourne Australia. https://informme.org.au/en/Guidelines/Clinical-Guidelines-for-Stroke-Management. 2019; Accessed 28 Aug 2019.

9. Kings College London for the Stroke Alliance for Europe. The Burden of stroke in Europe report. https://strokeeurope.eu/index/rehabilitation-and-long-term-support/5-4early-supported-discharge/ 2017; Accessed 28 Aug 2019.

10. Sentinel Stroke National Audit Programme (SSNAP). Post-acute organisational audit. Phase 2: Organisational audit of post-acute stroke service providers. https://www.strokeaudit.org/results/Organisational.aspx. 2015; Accessed 15 Mar 2019.

11. Saka O, Serra V, Samyshkin Y, McGuire A, Wolfe C. Cost-Effectiveness of Stroke Unit Care Followed by Early Supported Discharge. Stroke. 2009; 40:24-29.

12. Neale S, Leach K, Steinfort S, Hitch D. Costs and length of stay associated with early supported discharge for moderate and severe stroke survivors. Journal of Stroke and Cerebrovascular Diseases. 2020; 29:e104996. doi:10.1016/j.jstrokecerebrovasdis.2020.104996

13. Sentinel Stroke National Audit Programme. National Clinical results. https://www.strokeaudit.org/results/Clinical-audit/National-Results.aspx. 2018-2019; Accessed 8 Jun 2020.

14. Rudd AG, Hoffman A, Paley L, Bray B. 20 years of researching stroke through audit. Clin Rehabil. 2018; 32:997-1006.

15. Fisher RJ, Byrne A, Chouliara N, Lewis S, Paley L, Hoffman A, Rudd A, Robinson T, Langhorne P, Walker MF. Effectiveness of Stroke Early Supported Discharge: Analysis from a National Stroke Registry. Circulation: Cardiovascular Quality and Outcomes. 2020; (In press)

16. Fisher R, Chouliara N, Byrne A, Lewis S, Langhorne P, Robinson T, Waring J, Geue C, Hoffman A, Paley L, Rudd A, Walker MF. What is the impact of large-scale implementation of stroke Early Supported Discharge? A mixed methods realist evaluation study protocol. Implement Sci. 2019; 14:61.

For peer review only - http://bmjopen.bmj.com/site/about/guidelines.xhtml

17. NHS Midlands and East. NHS Midlands and East Stroke Service Specification. https://www.england.nhs.uk/mids-east/wp-content/uploads/sites/7/2018/03/final-stroke-spec.pdf. 2012; Accessed 15 Mar 2019.

18. Sentinel Stroke National Audit Programme. A Guide to Therapy Data. https://ssnap.zendesk.com/hc/en-us/articles/360001955854-Download-fullpack-of-Therapy-Report. 2017; Accessed 6 Mar 2019.

19. Sentinel Stroke National Audit Programme. National Clinical results. https://www.strokeaudit.org/results/Clinical-audit/National-Results.aspx. 2016; Accessed 6 Mar 2019.

20. Department of Health. Measures from the Adult Social Care Outcomes Framework, England. https://digital.nhs.uk/data-and-information/publications/statistical/adult-social-care-outcomes-framework-ascof. 2015-2016; Accessed 6 Mar 2019.

21. Turner M, Barber M, Dodds H, Murphy D, Dennis M, Langhorne P, Macleod MJ. Implementing a simple care bundle is associated with improved outcomes in a national cohort of patients with ischemic stroke. Stroke. 2015; 46:1065-70.

22. Teale E, Forster A, Munyombwe T, Young JB. A systematic review of case-mix adjustment models for stroke. Clin Rehabil. 2012; 26:771-786.

23. Raine R, Fitzpatrick R, Barratt H, Bevan G, Black N, Boaden R, Bower P, Campbell M, Denis JL, Devers K, Dixon-Woods M, Fallowfield L, Forder J, Foy R, Freemantle N, Fulop NJ, Gibbons E, Gillies C, Goulding L, Grieve R, Grimshaw J, Howarth E, Lilford RJ, McDonald R, Moore G, Moore L, Newhouse R, O'Cathain A, Or Z, Papoutsi C, Prady S, Rycroft-Malone J, Sekhon J, Turner S, Watson SI, Zwarenstein M. Challenges, solutions and future directions in the evaluation of service innovations in health care and public health. Health Serv Deliv Res. 2016; 4:16.

24. Morris S, Hunter RM, Ramsay AIG, Boaden R, McKevitt C, Perry C, Pursani N, Rudd AG, Schwamm LH, Turner SJ, Tyrrell PJ, Wolfe CDA, Fulop NJ. Impact of centralising acute stroke services in English metropolitan areas on mortality and length of hospital stay: difference-in-differences analysis. BMJ. 2014; 349:g4757.

25. Rafferty A, Walthery P, King-Hele S. Analysing Change Over Time: repeated cross sectional and longitudinal survey data. UK Data Service, University of Essex and University of Manchester. 2015.

26. Propensity Score Matching in Stata using teffects. https://www.ssc.wisc.edu/sscc/pubs/stata_psmatch.htm. 2015; Accessed 28 Nov 2019.

27. Sekhon, J. S. Multivariate and Propensity Score Matching Software with Automated Balance Optimization: The Matching package for R. Journal of Statistical Software. 2011; 42:1-52.

28. Sentinel Stroke National Audit Programme: Cost and Cost-effectiveness analysis. https://www.strokeaudit.org/SupportFiles/Documents/Health-Economics/Health-economic-report-2016.aspx. Accessed 8 Jun 2020.

For peer review only - http://bmjopen.bmj.com/site/about/guidelines.xhtml

29. Cobley CS, Fisher RJ, Chouliara N, Kerr M, Walker MF. A qualitative study exploring patients' and carers' experiences of Early Supported Discharge Services after stroke. Clin Rehabil. 2013; 27:750-757.

30. Chouliara N, Fisher RJ, Kerr M, Walker MF. Implementing evidence-based Stroke Early Supported Discharge services: a qualitative study of challenges, facilitators and impact. Clin Rehabil. 2014; 28:370-377.

31. Swinkels A, Mitchell T. Delayed transfer from hospital to community settings: the older person's perspective. Health Soc Care Community. 2008; 17:45-53.

32. Tistad M, von Koch L. Usual Clinical Practice for Early Supported Discharge after Stroke with Continued Rehabilitation at Home: An Observational Comparative Study. PLoS ONE. 2015; 10:e0133536. doi:10.1371/journal.pone.0133536

33. Bråndal1 A, Eriksson M, Glader EL, Wester P. Effect of early supported discharge after stroke on patient reported outcome based on the Swedish Riksstroke registry. BMC Neurology. 2019; 19:40. https://doi.org/10.1186/s12883-019-1268-8

34. Fisher RJ, Gaynor C, Kerr M, Langhorne P, Anderson C, Bautz-Holter E, Indredavik B, Mayo NE, Power M, Rodgers H, Rønning OM, Holmqvist LW, Wolfe CDA, Walker MF. A consensus on stroke: early supported discharge. Stroke. 2011; 42:1392-7.

35. Fisher RJ, Cobley CS, Potgieter I, Moody A, Nouri F, Gaynor C, Byrne A, Walker MF. Is Stroke Early Supported Discharge still effective in practice? A prospective comparative study. Clin Rehabil. 2016; 30:268-276.

36. Gittins M, Lugo-Palacios DG, Paley L, Bray B, Bowen A, Vail A, Gannon B, Tyson S. How do patients pass through stroke services? Identifying stroke care pathways using national audit data. Clin Rehabil. 2020; 34:698-709.

Figure Legends

Figure 1: Study design flow chart
SSNAP, Sentinel Stroke National Audit Programme; LOS, length of stay; RCS, repeated cross sectional; WISE, What is the Impact of Stroke Early supported discharge?; ESD, early supported discharge.

For peer review only

For peer review only - http://bmjopen.bmj.com/site/about/guidelines.xhtml

Tables

Table 1| Characteristics of 2015-2016 patient cohort

Patient Characteristics		ESD (n = 8648)	Non-ESD (n = 22908)
Age,y	<60	1422 (16.4)	3674 (16.0)
	60-69	1692 (19.6)	3932 (17.2)
	70-79	2646 (30.6)	6080 (26.5)
	80-89	2373 (27.4)	6873 (30.0)
	>89	515 (6.0)	2349 (10.3)
Gender	Male	4869 (56.3)	11785 (51.4)
Already inpatient at time of stroke	Yes	264 (3.1)	995 (4.3)
Congestive heart failure prior to admission	Yes	348 (4.0)	994 (4.3)
Hypertension prior to admission	Yes	4743 (54.9)	12299 (53.7)
Atrial fibrillation prior to admission	Yes	1370 (15.8)	4016 (17.5)
Diabetes prior to admission	Yes	1770 (20.5)	4834 (21.1)
Stroke/TIA prior to admission	Yes	2075 (24.0)	6100 (26.6)
Modified Rankin Scale score before stroke	0	5688 (65.8)	12796 (55.9)
	>0	2960 (34.2)	10112 (44.1)
NIHSS score on arrival	0	1041 (12.0)	3267 (14.3)
	1-5	4640 (53.7)	11252 (49.1)

For peer review only - http://bmjopen.bmj.com/site/about/guidelines.xhtml

	6-14	2253 (26.1)	5596 (24.4)
	15-24	636 (7.4)	2322 (10.1)
	>24	78 (0.9)	471 (2.1)
Type of stroke	Infarction	7804 (90.8)	20772 (91.2)
	Primary Intracerebral Haemorrhage	789 (9.2)	2002 (8.8)
Modified Rankin Scale score at inpatient discharge	0	1046 (12.1)	4016 (17.5)
	1	2259 (26.1)	5024 (21.9)
	2	1928 (22.3)	3420 (14.9)
	3	2189 (25.3)	3838 (16.8)
	4	1118 (12.9)	4154 (18.1)
	5	108 (1.3)	2456 (10.7)
Median (IQR) total length of hospital stay in days		6.9 (2.9-18.8)	6.0 (2.2-24.8)

Values are numbers (percentages) unless stated otherwise. NIHSS indicates National Institutes of Health Stroke Scale; TIA, transient ischemic attack; and IQR, interquartile range.

Table 2| Association between having received ESD on the care pathway and inpatient length of stay 2015-2016

Inpatient length of stay models	Unadjusted		Adjusted*	
Patients	30975		30791	
Admitting hospitals	55		55	
Patients per admitting hospital: Min	27		26	
Mean	563.2		559.8	
Max	1331		1310	
Intraclass correlation coefficient	0.05		0.08	
**ESD on the care pathway**	**Coefficient (95% CI)**	**P value**	**Coefficient (95% CI)**	**P value**
Received ESD	0.08 (0.04 to 0.12)	< 0.001	0.15 (0.12 to 0.18)	< 0.001

Model coefficients are on the natural log scale; significant results were back-transformed, that is, exponentiated to obtain percentage change in length of hospital stay (reported in text). ESD indicates early supported discharge; NIHSS, National Institutes of Health Stroke Scale; ASCOF, Adult Social Care Outcomes Framework; and SSNAP, Sentinel Stroke National Audit Programme.

** Adjusted for age, sex, prestroke independence, comorbidities, NIHSS score on admission, type of stroke and modified Rankin score at discharge from hospital (patient level); ASCOF rate and hospital SSNAP rating score (admitting hospital level)*

For peer review only

For peer review only - http://bmjopen.bmj.com/site/about/guidelines.xhtml

Table 3| Matched analysis between patients who received ESD on the care pathway and similar patients who did not 2015-2016+

**Inpatient matching models**	**Between***		**Within***	
Patients	30791 (8593 ESD + 22198 non-ESD)		10449 (4572 ESD + 5877 non-ESD)	
Admitting hospitals	55		14	
**ESD on the care pathway**	**Coefficient (95% CI)**	**P value**	**Coefficient (95% CI)**	**P value**
Received ESD	0.07 (0.03 to 0.11)	< 0.001	0.08 (0.05 to 0.12)	< 0.001

Model coefficients are on the natural log scale; significant results were back-transformed, that is, exponentiated to obtain percentage difference in length of hospital stay (reported in text). ESD indicates early supported discharge; and NIHSS, National Institutes of Health Stroke Scale.

+ This is a sensitivity analysis using propensity score matching to complement the multilevel model results presented in Table 2. Between means patients in all hospitals were matched even if the hospitals did not offer ESD whereas Within means only hospitals with patients who did and did not receive ESD were matched

** Adjusted for age, sex, prestroke independence, comorbidities, NIHSS score on admission, type of stroke and modified Rankin score at discharge from hospital (patient level)*

For peer review only - http://bmjopen.bmj.com/site/about/guidelines.xhtml

Table 4| Patient characteristics from 2013-2014 & 2015-2016 cohorts

Patient Characteristics		ESD (n = 11376)	Non-ESD (n = 39417)
Age,y	<60	1847 (16.2)	6212 (15.8)
	60-69	2239 (19.7)	6647 (16.9)
	70-79	3467 (30.5)	10543 (26.8)
	80-89	3126 (27.5)	12087 (30.7)
	>89	697 (6.1)	3928 (10)
Gender	Male	6352 (55.8)	19959 (50.6)
Already inpatient at time of stroke	Yes	361 (3.2)	1766 (4.5)
Congestive heart failure prior to admission	Yes	481 (4.2)	1558 (4)
Hypertension prior to admission	Yes	6261 (55)	21313 (54.1)
Atrial fibrillation prior to admission	Yes	1840 (16.2)	7299 (18.5)
Diabetes prior to admission	Yes	2345 (20.6)	8074 (20.5)
Stroke/TIA prior to admission	Yes	2740 (24.1)	10482 (26.6)
Modified Rankin Scale score before stroke	0	7389 (65)	22847 (58)
	>0	3987 (35.1)	16570 (42)
NIHSS score on arrival	0	1430 (12.6)	6884 (17.5)
	1-5	6020 (52.9)	18802 (47.7)
	6-14	2955 (26)	9162 (23.2)
	15-24	861 (7.6)	3833 (9.7)
	>24	110 (1)	736 (1.9)
Type of stroke	Infarction	10315 (91.2)	35989 (92.1)
	Primary Intracerebral Haemorrhage	998 (8.8)	3079 (7.9)
Modified Rankin Scale score at inpatient discharge	0	1440 (12.7)	8474 (21.5)
	1	2928 (25.7)	8649 (21.9)
	2	2562 (22.5)	5456 (13.8)
	3	2817 (24.8)	6090 (15.5)
	4	1471 (12.9)	6733 (17.1)

For peer review only - http://bmjopen.bmj.com/site/about/guidelines.xhtml

	5	158 (1.4)	4015 (10.2)
Time period	2013-2014	3065 (26.9)	21841 (55.4)
	2015-2016	8311 (73.1)	17576 (44.6)
Median (IQR) total length of hospital stay in days			
	2013-2014	7.7 (3.2-23.8)	6.3 (2.5-22.1)
	2015-2016	6.8 (2.9-18.5)	5.7 (2.1-24.6)

Values are numbers (percentages) unless stated otherwise. NIHSS indicates National Institutes of Health Stroke Scale; TIA, transient ischemic attack; and IQR, interquartile range.

Table 5| Association between having received ESD on the care pathway and inpatient length of stay 2013-2016

Inpatient length of stay models		Unadjusted		Adjusted*	
Patients		49667		49266	
Admitting hospitals		41		41	
Patients per admitting hospital:	Min	115		106	
	Mean	1211.4		1201.6	
	Max	2512		2446	
Intraclass correlation coefficient		0.04		0.08	
**ESD on the care pathway**		**Coefficient (95% CI)**	**P value**	**Coefficient (95% CI)**	**P value**
Received ESD		0.14 (0.09 to 0.20)	< 0.001	0.17 (0.13 to 0.22)	< 0.001
2015-2016 time period		-0.06 (-0.09 to -0.04)	< 0.001	-0.15 (-0.17 to -0.13)	< 0.001
Received ESD * 2015-2016 time period		-0.05 (-0.11 to 0.02)	0.162	0.01 (-0.04 to 0.06)	0.790

Model coefficients are on the natural log scale; significant results were back-transformed, that is, exponentiated to obtain percentage change in length of hospital stay (reported in text). ESD indicates early supported discharge; NIHSS, National Institutes of Health Stroke Scale; ASCOF, Adult Social Care Outcomes Framework; and SSNAP, Sentinel Stroke National Audit Programme.

** Adjusted for age, sex, prestroke independence, comorbidities, NIHSS score on admission, type of stroke and modified Rankin score at discharge from hospital (patient level); ASCOF rate and hospital SSNAP rating score (admitting hospital level)*

For peer review only - http://bmjopen.bmj.com/site/about/guidelines.xhtml

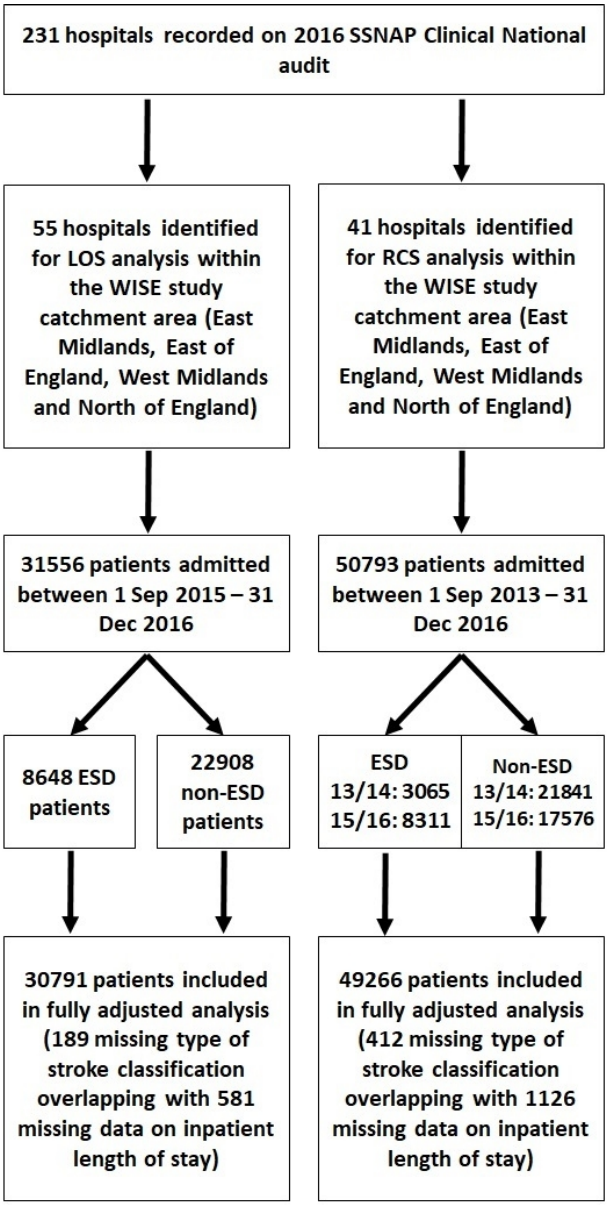

Figure 1: Study design flow chart
SSNAP, Sentinel Stroke National Audit Programme; LOS, length of stay; RCS, repeated cross sectional; WISE, What is the Impact of Stroke Early supported discharge?; ESD, early supported discharge.

138x273mm (96 x 96 DPI)

STROBE Statement—Checklist of items that should be included in reports of ***cross-sectional studies***

	Item No	Recommendation	Page No
**Title and abstract**	1	(*a*) Indicate the study's design with a commonly used term in the title or the abstract	0,1
		(*b*) Provide in the abstract an informative and balanced summary of what was done and what was found	1
**Introduction**			
Background/rationale	2	Explain the scientific background and rationale for the investigation being reported	3
Objectives	3	State specific objectives, including any prespecified hypotheses	4
**Methods**			
Study design	4	Present key elements of study design early in the paper	4,5
Setting	5	Describe the setting, locations, and relevant dates, including periods of recruitment, exposure, follow-up, and data collection	5
Participants	6	(*a*) Give the eligibility criteria, and the sources and methods of selection of participants	5
Variables	7	Clearly define all outcomes, exposures, predictors, potential confounders, and effect modifiers. Give diagnostic criteria, if applicable	6,7
Data sources/ measurement	8*	For each variable of interest, give sources of data and details of methods of assessment (measurement). Describe comparability of assessment methods if there is more than one group	5
Bias	9	Describe any efforts to address potential sources of bias	8
Study size	10	Explain how the study size was arrived at	4
Quantitative variables	11	Explain how quantitative variables were handled in the analyses. If applicable, describe which groupings were chosen and why	6,7
Statistical methods	12	(*a*) Describe all statistical methods, including those used to control for confounding	7
		(*b*) Describe any methods used to examine subgroups and interactions	8
		(*c*) Explain how missing data were addressed	8
		(*d*) If applicable, describe analytical methods taking account of sampling strategy	7
		(*e*) Describe any sensitivity analyses	9
**Results**			
Participants	13*	(a) Report numbers of individuals at each stage of study—eg numbers potentially eligible, examined for eligibility, confirmed eligible, included in the study, completing follow-up, and analysed	10,11
		(b) Give reasons for non-participation at each stage	N/A
		(c) Consider use of a flow diagram	4
Descriptive data	14*	(a) Give characteristics of study participants (eg demographic, clinical, social) and information on exposures and potential confounders	10,11
		(b) Indicate number of participants with missing data for each variable of interest	N/A
Outcome data	15*	Report numbers of outcome events or summary measures	10,11
Main results	16	(*a*) Give unadjusted estimates and, if applicable, confounder-adjusted estimates and their precision (eg, 95% confidence interval). Make clear which confounders were adjusted for and why they were included	21,24

For peer review only - http://bmjopen.bmj.com/site/about/guidelines.xhtml

		(*b*) Report category boundaries when continuous variables were categorized	20,23
		(*c*) If relevant, consider translating estimates of relative risk into absolute risk for a meaningful time period	N/A
Other analyses	17	Report other analyses done—eg analyses of subgroups and interactions, and sensitivity analyses	10,22
**Discussion**			
Key results	18	Summarise key results with reference to study objectives	12,13
Limitations	19	Discuss limitations of the study, taking into account sources of potential bias or imprecision. Discuss both direction and magnitude of any potential bias	13,14
Interpretation	20	Give a cautious overall interpretation of results considering objectives, limitations, multiplicity of analyses, results from similar studies, and other relevant evidence	14
Generalisability	21	Discuss the generalisability (external validity) of the study results	N/A
**Other information**			
Funding	22	Give the source of funding and the role of the funders for the present study and, if applicable, for the original study on which the present article is based	15

*Give information separately for exposed and unexposed groups.

Note: An Explanation and Elaboration article discusses each checklist item and gives methodological background and published examples of transparent reporting. The STROBE checklist is best used in conjunction with this article (freely available on the Web sites of PLoS Medicine at http://www.plosmedicine.org/, Annals of Internal Medicine at http://www.annals.org/, and Epidemiology at http://www.epidem.com/). Information on the STROBE Initiative is available at www.strobe-statement.org.

For peer review only

For peer review only - http://bmjopen.bmj.com/site/about/guidelines.xhtml
